# Supplementary material for: Intervening to prevent a suicide in a public place: a qualitative study of effective interventions by lay people
Source: BMJ Open. 2019 Nov 18;9(11):e032319. doi: 10.1136/bmjopen-2019-032319 (PMC6887022; doi:10.1136/bmjopen-2019-032319)
Supplement: Supplementary data [file bmjopen-2019-032319supp001.pdf]

STRANGER ON THE BRIDGE: Topic guide for Group 1 'Survivors', Version 1; 16 Jan 2017

Topic guide
Group 1: Those with experience of being stopped from taking their own lives

Checklist:
- Information sheet and consent form
- Tape recorder
- Spare batteries
- Biscuits or cake
- Turn phone to silent

Preamble:
- *Thanks for coming forward; importance of understanding the situation from your point of view*
- *Brief recap of aims of study*
- *Recap of confidentiality*
- ***Ask directly*** *about current mental state and suicidality: "Have you had any thoughts of suicide recently [in the last week or two]? How are you feeling right now?" If currently receiving psychiatric care, do they have a letter from psychiatrist? If in any doubt, advise not to proceed.*
- *Stopping rule: "I realise that talking about that time will be difficult for you. You are completely free to stop anytime you wish, or you can take a break and then decide whether or not to carry on. Please tell me if talking about it triggers thoughts of harming yourself or makes you feel in any way unsafe. If that happens, you* ***must*** *stop."*
- *Sign consent form*
- *Brief outline of interview*

Opening free narrative:

"So I know that you came close to taking your own life in [month/year]. In your own time, please tell me what led up to it and what happened on that particular day."

Prompts and suggested follow-up. Ensure the following are fully explored:

Setting:
- Clarify where exactly it took place
- Why there (choice of location)? Why that method of suicide? Had you thought about other places and possibly other methods of ending your life? How carefully planned was it?
- How did you get there? How long did the journey take?
- What was going through your mind when you got there? [Probe: thinking and feeling]

Intent:
- How much did you want to die?
  or
  Did you think someone might try to stop you? Was any part of you hoping that might happen?

Intervention:
- Who approached you and how?
- What was the first thing they said to you? Then what…?
- How did they say it? [Probe: body language, positioning, eye contact, touch, other non-verbal signals, pace and timing, general manner and tone of voice.
- How did it make you feel?

STRANGER ON THE BRIDGE: Topic guide for Group 1 'Survivors', Version 1; 16 Jan 2017

- What other actions did they take, including calling emergency services?
- What helped most, made the most difference?
- What wasn't helpful, irritated or angered you?
- What sort of things should people avoid saying or doing?

- What message would you like to give to anyone who is unsure about whether or not to intervene?

- Thinking more broadly about prevention and about that particular place, apart from someone stepping in and talking to you, is there anything else that might have prevented you from going through with the suicide? [Prompt if necessary: e.g. higher parapet on bridge, fencing on railways, emergency telephone…]

Participant's background

- Age
- Ethnicity
- Region (London; South East; South West etc)
- Employment status [employed; unemployed; student; homemaker; carer; retired…]
- If working, what do they do?

- Have you kept in touch with the person who helped you? If so, how would you feel about us possibly contacting them and asking them if they would be interviewed?

- Is there anything else you'd like to add?

Close:

- *Thanks again etc.*
- *Offer Amazon voucher and check email address*
- *Tell them they can get in touch if they think of anything afterwards that they'd like to add or change*
- *Ask if they'd like to receive further information about the study and a summary of the findings*
- *Ask if they know others who've been in similar situation who might want to take part*
- *Ask if they have any ideas about how to advertise study.*

Debrief/safety check:

- *How are they feeling right now? **Ask directly**: "Has talking about that day made you feel like harming yourself again?"*
- *Do they have plans for the rest of the day? Where are they planning to go [immediately after the interview]; are they meeting up with anyone? Offer to stay with them until then, or ask them to text you when they get there*
- *Give details of organisations to contact for support if they become upset later*
- *If they give any cause for concern, ask if you can accompany them to a place of safety (a trusted friend's house or A&E).*
