# Supplementary material for: Intervening to prevent a suicide in a public place: a qualitative study of effective interventions by lay people
Source: BMJ Open. 2019 Nov 18;9(11):e032319. doi: 10.1136/bmjopen-2019-032319 (PMC6887022; doi:10.1136/bmjopen-2019-032319)
Supplement: Supplementary data [file bmjopen-2019-032319supp002.pdf]

STRANGER ON THE BRIDGE: Topic guide for Group 2 'Interveners', Version 1; 16 Jan 2017

Topic guide
Group 2: Those with experience of intervening

Checklist:
- Consent form
- Tape recorder
- Spare batteries
- Biscuits or cake
- Turn phone to silent

Preamble:
- *Thanks for coming forward; importance of understanding the situation from your point of view*
- *Brief recap of aims of study*
- *Recap of confidentiality*
- *Possible that it may be upsetting to recall what happened. If that happens, please tell me and we will stop the interview. You are completely free stop anytime you wish, or you can take a break and then decide whether or not to carry on.*
- *Sign consent form*
- *Brief outline of interview*

Opening free narrative:

"So I know that in [month/year] you saw someone in a public place who you thought was at risk of harming themselves and that you tried to help them. In your own time, please tell me what happened on that particular day."

Prompts and suggested follow-up. Ensure the following are fully explored:

Setting
- Clarify where exactly it took place
- What exactly were you doing when you first noticed the person? Where were you going? Paint a picture of the scene.

Recognition
- What was the first thing you noticed about them?
- How quickly did you realise that they might be in need of help of some sort?
- Did you immediately recognise that they were at risk of harming themselves?
- What was it that enabled you to know that? [Probe: features, appearance, behaviour of suicidal person **plus** participant's own background /previous experience or exposure]

Preparedness
- What went through your mind when you realised that?
- How quickly did you decide to approach them, and how did you make that decision?
- Did you know what to say or do, or were you just 'winging it'?
- How confident did you feel that you could help them? [Probe for: fears about intervening; feelings about competence]

STRANGER ON THE BRIDGE: Topic guide for Group 2 'Interveners', Version 1; 16 Jan 2017

<u>Intervention</u>

- What was the first thing you said or did?
- Then what…?
- What was going through your mind while it was all happening? How were you feeling?
- Do you think you made any mistakes? Anything you regret saying or doing? If so, what?
- What would you do differently another time?
- Do you have any thoughts about what was most helpful?

<u>Personal consequences:</u>

- What impact has it had on you? [<u>Probe:</u> immediate; longer-term, depending on how long ago; future]
- Would you do it again? Why/why not?
- What message would you give to anyone who is unsure about whether or not to intervene in a situation like the one you encountered?

<u>Training</u>

- <u>If not already mentioned:</u> Have you received any mental health or suicide prevention training? If so, what and when?
- Do you think members of the public should receive basic suicide prevention training, in the same way that they get first aid training? What form do you think it should take?
- What sort of information would have been helpful to you in the situation you found yourself in?

<u>Participant's background</u>

- Age
- Ethnicity
- Region (London; South East; South West etc)
- Employment status [employed; unemployed; student; homemaker; carer; retired…]
- If working, what do they do?

- Have you kept in touch with the person you helped? If so, how would you feel about us possibly contacting them and asking them if they would be interviewed?

- Is there anything else you'd like to add?

<u>Close:</u>

- *Thanks again etc.*
- *Offer Amazon voucher and check email address*
- *Tell them they can get in touch if they think of anything afterwards that they'd like to add or change*
- *Ask if they'd like to receive further information about the study and a summary of the findings*
- *Ask if they have any links with others in similar situation*
- *Ask if they have any ideas about how to advertise the study.*

<u>Safety check:</u>

- *How are they feeling right now? Has it been upsetting recalling the events of that day?*
- *If so, check plans for the rest of the day? Do they have someone they can meet up with?*
- *Give details of organisations to contact for support*
