# Supplementary material for: Intervening to prevent a suicide in a public place: a qualitative study of effective interventions by lay people
Source: BMJ Open. 2019 Nov 18;9(11):e032319. doi: 10.1136/bmjopen-2019-032319 (PMC6887022; doi:10.1136/bmjopen-2019-032319)
Supplement: Author's manuscript [file bmjopen-2019-032319.draft_revisions.pdf]

BMJ Open

BMJ Open is committed to open peer review. As part of this commitment we make the peer review history of every article we publish publicly available.

When an article is published we post the peer reviewers' comments and the authors' responses online. We also post the versions of the paper that were used during peer review. These are the versions that the peer review comments apply to.

The versions of the paper that follow are the versions that were submitted during the peer review process. They are not the versions of record or the final published versions. They should not be cited or distributed as the published version of this manuscript.

BMJ Open is an open access journal and the full, final, typeset and author-corrected version of record of the manuscript is available on our site with no access controls, subscription charges or pay-per-view fees (http://bmjopen.bmj.com).

If you have any questions on BMJ Open's open peer review process please email info.bmjopen@bmj.com

BMJ Open

Preventing suicides in public places: A qualitative interview study to identify the core components of an effective intervention by a member of the public

Journal:	*BMJ Open*
Manuscript ID	bmjopen-2019-032319
Article Type:	Research
Date Submitted by the Author:	12-Jun-2019
Complete List of Authors:	Owens, Christabel ; University of Exeter, College of Medicine and Health<br>Derges, Jane; University of Exeter, College of Medicine and Health<br>Abraham, Charles; University of Melbourne, School of Psychological Sciences
Keywords:	Suicide & self-harm < PSYCHIATRY, PUBLIC HEALTH, QUALITATIVE RESEARCH, MENTAL HEALTH

SCHOLARONE™
Manuscripts

Preventing suicides in public places:

A qualitative interview study to identify the core components of an effective intervention by a member of the public

For peer review only

Authors:

Christabel Owens,* University of Exeter Medical School, Exeter, UK

Jane Derges, University of Exeter Medical School, Exeter, UK

Charles Abraham, School of Psychological Sciences, University of Melbourne, Australia

*** Corresponding author:**

Professor Christabel Owens, University of Exeter Medical School, College House, St Luke's Campus, Exeter EX1 2LU, United Kingdom.

Tel: +44 (0)1392 726006

Email: c.v.owens@exeter.ac.uk

ABSTRACT

Objectives: Many suicides take place in public locations, usually involving jumping from high places or use of transport networks. Previous research has focused on the effectiveness of physical barriers at frequently-used locations. There have been no studies of human intervention to prevent suicides in public places. The aim of this study was to identify the core components of an effective intervention by a member of the public.

Design and methods: We conducted in-depth qualitative interviews with people who have either been prevented by a stranger from taking their own life in a public location (n=12), or intervened to prevent a stranger from taking their own life in a public location (n=21). Collectively, the two groups narrated 50 incidents of suicide rescue. We analysed interview transcripts using inductive thematic analysis.

Results: Suicidal people typically displayed no visible distress, describing themselves as being dissociated or "in a bubble". Intervention was seen to involve three main tasks: "Bursting the bubble" (reconnecting with self, others and everyday world); Moving to a safer location, and Summoning help. We show how interveners accomplished these tasks in a range of ways, using both verbal and non-verbal communication. The data suggest that what the intervener said was much less important than how they made the suicidal person feel, namely safe, connected and validated ("I matter").

Conclusions:

This is the first empirical study to examine the role of passing strangers in preventing suicides in public places and provides clear evidence on which to base guidance for

For peer review only - http://bmjopen.bmj.com/site/about/guidelines.xhtml

members of the public. It shows that no specialist skills and no script are needed. The situation calls for openness, authenticity, and compassion. A set of simple public education messages is needed to empower people to recognise and reach out to those who may be at risk of suicide in public locations.

For peer review only

For peer review only - http://bmjopen.bmj.com/site/about/guidelines.xhtml

STRENGTHS AND LIMITATIONS OF THIS STUDY

- This is the first empirical study to examine the role of passing strangers in preventing suicides.
- We included the perspectives of both 'rescuers' and 'rescued' and found a very high level of agreement between them as to what worked, giving confidence in our conclusions about effective interventions.
- Although we did not specify that interventions must have been successful, our intervener group did not include any individuals who had tried and failed to avert a suicide, so we are unable to draw any conclusions about ineffective interventions.
- We allowed a wide range in the length of time between incident and interview (from less than one year to more than ten years), extending our original upper limit of five years in some cases after careful screening to assess the quality of recall; all participants were able to give extremely detailed accounts, which we had no reason to doubt.
- Our model of effective suicide crisis intervention may be culturally-specific and may not be suited to other countries, particularly those in east Asia that have much higher incidence of suicides in public places.

For peer review only - http://bmjopen.bmj.com/site/about/guidelines.xhtml

INTRODUCTION

Suicide accounts for around 6,000 UK deaths each year, up to a third of which occur in public locations.[1] These may be more easily preventable than those that occur in the privacy of the home, because of the potential for a chance passer-by to make a last-minute intervention. There is compelling anecdotal evidence that lives can be saved by passing strangers acting on the spur of the moment. *The Stranger on the Bridge* was an award-winning documentary, broadcast on UK television in 2015, which told the story of one such intervention and captured global media attention.[2]

Preventing suicides in public places occupies a key place in England's national suicide prevention strategy.[3] In 2015 Public Health England published a practice resource for local public health teams to help them identify sites and structures that might be used for suicides and take action to prevent deaths at those locations.[4] These typically include sites that provide opportunity for jumping from a height (bridges, high buildings and cliffs) and sections of the road and railway networks. Previous research has largely focused on the effectiveness of installing physical barriers at such locations to restrict access to means.[5-7] There are no studies of human intervention, and no evidence-based guidance for members of the public. The literature contains only one published article on the topic of what a potential helper might say in a 'Stranger on the Bridge' scenario.[8] Based on the authors' clinical experience rather than research evidence, it presents a suggested script that is long, verbose and contains no clear messages for the general public; nor has it been tested. The World Health Organization (WHO) has published guidance for emergency services personnel responding to suicidal crises,[9] but the advice is very general and does not differentiate between crises occurring in private and in public places. There is an extensive literature on police crisis negotiation, some of

For peer review only

which deals with suicide intervention,[10-12] but this is either too general, in the manner of the WHO document, or too specialized, describing 'tricks of the trade' that could not be safely recommended to lay people.

We were not interested in the part played by health professionals or members of the emergency services, whose jobs include suicide crisis intervention, but in ordinary citizens who happen to be passing by. The aim of this study was to identify the core components of an effective intervention by a member of the public in a 'Stranger on the Bridge' situation.

For peer review only

For peer review only - http://bmjopen.bmj.com/site/about/guidelines.xhtml

METHODS

Design

We conducted a qualitative interview study in order to understand intervention by a passer-by from the perspective of both 'rescuers' and 'rescued'.

Participants and sampling

Participants were eligible for inclusion if they were aged 18 or over and had experience of either of the following:

- Being stopped from taking their own life in a public location by a passing stranger (Rescued group);
- Trying to stop someone they did not know from taking their own life in a public location (Intervener group). We intended that this group would include members of the general public and members of staff in non-health agencies whose work at public locations brings them into contact with suicidal individuals, such as railway staff.

A public location was defined as anywhere outside their own or someone else's home. For both groups, we placed advertisements on the websites of mental health and suicide prevention charities and used the social media accounts (Facebook and Twitter) of non-academic partners to invite people with relevant experience to come forward. Group 2 participants were also sought via relevant agencies, including Network Rail, Highways England and bridge authorities. We continued recruiting until each group contained a reasonable degree of diversity in respect of age, gender and ethnicity and we were confident that no new insights were emerging (data saturation).

Interviews

For peer review only - http://bmjopen.bmj.com/site/about/guidelines.xhtml

We conducted in-depth interviews with both groups, beginning with an invitation to give an uninterrupted account: "Please tell me in your own words as much as you can remember about [the event in question]." This free narrative approach affords insight into what is uppermost in the participant's mind.[13-15] It also allows them to disclose information at their own pace, thereby increasing their sense of safety when talking about difficult or sensitive subjects.[16, 17] During the narrative phase the interviewer [JD] assumed the role of active listener. She then proceeded to ask open-ended questions, following up any points requiring clarification and probing for further detail where necessary.[18] Separate interview guides for each group were used to ensure that all topics of interest were covered and that safety protocols were followed. Interveners reflected in detail on what they said/did and how key decisions were made. Those with experience of being rescued described what the intervener said/did and how it affected them, allowing us to examine mechanisms of action.

All interviews with the rescued group were conducted face-to-face, mostly in participants' homes, and lasted 1-2 hours. Interviews with interveners were conducted face-to-face (n=15), by telephone (n=4) or Skype (n=2). All participants gave informed consent to audio-recording. Interviews were transcribed verbatim and anonymised.

Analysis

Data collection and analysis were carried out concurrently to allow for progressive focusing of interviews and exploration of emerging ideas. Two investigators [CO and JD] independently read the first three transcripts from each group (n=6) before meeting to discuss candidate themes and analytic strategy. They then worked line-by-line through six further transcripts, noting and naming specific features of the situation, including:

For peer review only - http://bmjopen.bmj.com/site/about/guidelines.xhtml

characteristics of the actors; discrete intervention elements (verbal and non-verbal) and their effects on the suicidal person; constraints, complicating factors and tipping points. These were used to construct a list of categories, with definitions, that was used for coding the entire data set. Coding, sorting and retrieval were carried out using NVivo software. We then carefully examined all the coded material, exploring patterns within and relationships between categories, visually mapping intervention elements, and working via a series of iterations and data checks towards our final interpretive model.

Patient and public involvement

We formulated the question and designed the study in consultation with people with relevant lived experience, representatives from suicide prevention charities and the transport industry. They were instrumental in recruiting participants and subsequently commenting on findings and interpretation. We will continue to work with them as we start to frame key public education messages. A lay summary of results will be sent to participants, who will also be invited to help with future message development work.

Ethical approval

Ethical approval was granted by the [institution] Research Ethics Committee.

For peer review only

RESULTS

Characteristics of the sample

We recruited 12 people who had been stopped from taking their own life in a public place (Rescued): nine female and three male, with ages ranging from 19-64. Four of them had been rescued on more than one occasion (one twice and three on three occasions), giving a total of 19 accounts of successful intervention.

The Intervener group contained 21 individuals: nine male and twelve female, also ranging in age from 19-64. This number was made up of 13 members of the general public, six railway workers[a] (two of whom were off-duty at the time) and two highways officers. Between them they had conducted 31 interventions, all successful. Most of the railway workers and one of the highways officers had intervened successfully on more than one occasion. Three members of the public had conducted more than one rescue. One participant was included in both groups because, having been stopped from taking her own life in a public place, she went on to conduct two successful interventions. The members of the public included students, teachers/lecturers, youth leaders, church and charity workers, civil servants, office workers and an actor. Several of them had lived experience of mental health problems. They recounted how, during the intervention, they drew on a miscellany of skills and scraps of knowledge that they had acquired in other spheres, including work and leisure pursuits. One used the metaphor of an 'experience

[a] The railway staff were all Mobile Operations Managers, whose role is to deal with a range of incidents on the railway network, including threatened suicides and fatalities. They differed from members of the general public insofar as: a) they were often acting on a referral, so had some time to prepare; b) five out of the six had received basic suicide intervention training from Samaritans; c) they had responsibilities beyond caring for the suicidal person, including restoring train services as quickly as possible, and d) they had organisational arrangements in place for managing such incidents, including direct links to the British Transport Police. Our rationale for including them in the intervener group was that they were responding as 'lay people', i.e. not as mental health professionals, to a threat of suicide by a stranger in a public location.

For peer review only - http://bmjopen.bmj.com/site/about/guidelines.xhtml

bag' that she carried around with her and had raided for ideas and possible strategies to use while struggling on two different occasions to keep a suicidal person safe.

Of the 50 interventions recounted (19 by Rescued and 31 by Interveners), 27 occurred on the rail and road networks (including bridges over road or railway). A further 18 involved other types of bridge or high places. Characteristics of participants and settings are shown in Table 1.

Table 1: Participants and settings

	Rescued group (n=12)	Intervener group (n=21)
Age range	19-64	19-64
Gender:		
Female	9	12
Male	3	9
Ethnicity:		
White British/European	10	21
Asian/Asian British	2	-
Black/Black British	-	-
Other ethnic groups	-	-
Mixed/Multiple ethnicity	-	-
Location of rescue:	(n of incidents =19)	(n of incidents =31)
Rail/underground/bridge over railway	4	14
Road network/bridge over road	2	8
River/bridge over river	7	4
High building	3	0
Cliffs	2	3
Other	1	2
Time since incident:	(n=19)	(n=31)
1 year or less	7	7
2 – 5 years	8	10
6 - 10 years	2	12
More than 10 years	2	2

We present our findings under the following headings: Recognition; Three intervention tasks (Bursting the bubble, Moving to a safer location, and Summoning help), and Endings and aftermath.

For peer review only - http://bmjopen.bmj.com/site/about/guidelines.xhtml

Recognition

The prerequisite for intervention is recognition. Interveners recognised the person as being at risk or in need of help primarily by their location. The person either appeared to have placed themselves in immediate physical danger or was judged to be out of place, giving rise to curiosity or concern:

> Intervener (2.06):"There was a person standing there in a hoodie, sort of facing the railway and looking over the side … and I took a couple of steps and thought, Hang on, what's she doing there? I did literally stop and think, Hang on, this is odd."

Participants who had been rescued also acknowledged the oddness of their positioning:

> Rescued (1.01):"I went very close to the edge of the platform and sat with my feet over the edge … quite hunched up, possibly my hands over my ears and my eyes closed. I mean, it's not generally a position people take when they're waiting for a train."

There were few clear behavioural clues and rarely anything in their mode of dress or appearance to mark them out. What was most striking in interveners' accounts of what they saw was the absence of visible emotion. They described the suicidal person as looking "vacant", "glazed over", "zoned out" or "as if no-one was at home", but not distressed.[b] This was corroborated in the accounts of rescued participants, who

[b] There were two instances recounted by interveners in which the person was sobbing inconsolably. In both cases the person spoke of wanting to end their life, but neither was in a high-risk location or in possession of a means of suicide, suggesting that they were not about to carry out a plan. This was confirmed by a rescued participant (1.01), who said, *"I wouldn't even be able to walk to the station if I was crying that much. It would be when I've run out of tears … and I just feel empty and numb and disconnected. That's when I'm much more likely to act."*

For peer review only - http://bmjopen.bmj.com/site/about/guidelines.xhtml

described themselves as having moved into a space beyond emotion: "a weird sort of surreal, unfeeling state" in which they were "completely numb", "frozen" or "dissociated", cut off from themselves, others and the everyday world, to the point where they believed they were invisible. They were not inviting rescue:

> Rescued (1.11): "It didn't occur to me that people would stop and take notice. I was very much of this mindset that I was invisible and no-one would see me."

This state of dissociation was described as being "in a bubble".

Interveners, on the other hand, appear to have been characterised by a state of openness to what was going on around them and an attunement to human distress even when it was not manifest. Some reflected that this had been acquired from parents with a strong sense of social justice or through experience in particular settings, such as working as a bar manager: "They teach you to spot trouble before it happens" (Intervener 2.15). This participant had been walking across a bridge with his partner when they encountered a person preparing to take her own life. He knew immediately that something was amiss, whilst his partner was unaware of anything until he started to intervene.

Three intervention tasks

We identified three main tasks involved in intervention:

i) Bursting the bubble (reconnecting with self, others and the everyday world);

ii) Moving to a safer location;

iii) Summoning help.

For peer review only - http://bmjopen.bmj.com/site/about/guidelines.xhtml

The three tasks are interconnected, as shown in Figure 1 and described below. Interventions were typically complex and interveners' accounts, particularly those of general public members who did not have organisational structures around them, show them to be frantically multi-tasking during the course of the encounter, trying to think ahead like chess players and plan several possible moves. Occasionally they managed to achieve several purposes through a single speech act or gesture.

[INSERT FIGURE 1]

The order in which they approached the tasks and the manner of intervention was influenced by a number of factors, including: the immediacy of danger; the clarity of suicidal intent; the capacity of the suicidal person to interact verbally; the intervener's personality, and whether they were in a position of authority and responsibility. Interventions and their constituent parts fell on a spectrum according to the degree of control assumed by the intervener (Figure 2). At one end were interventions involving forcible restraint and authoritative language, often necessitated by the perceived urgency of the situation. At the opposite end, some interveners were acutely conscious of the suicidal person's right to self-determination and need to feel in control of their own destiny. Accordingly, they asked permission before taking any action and used invitations and appeals, calling on the person to rescue themselves and allowing them to keep all their options open:

> Rescued (1.05): "He came up behind and he said, Look, I don't want to stop you from doing anything but I'd like it if you could come over the barrier … just step on over, it's OK … And he said, I'm not going to take you away from here, you can stay here if you

For peer review only - http://bmjopen.bmj.com/site/about/guidelines.xhtml

want ... It was like, he's not forcing me to do anything. I can still do it ... and that was important for me."

[INSERT FIGURE 2]

i) Bursting the bubble

Both rescued and rescuers used the metaphor of the bubble and described intervention in terms of bringing the suicidal person out of their trance-like, dissociated state and back into the present moment, reconnecting them with themselves, others and the everyday world. This emotional labour formed the heart of most of the interventions we studied. It was predominantly verbal in nature, although embodied communications sometimes also played a key part.[c]

Initial approach: The work began with the intervener establishing their presence, usually with a verbal overture in the form of a question. Most common was, "Are you OK?" This apparently simple enquiry was favoured by interveners who were fearful of intrusion or confrontation, or unsure whether or not they should be concerned. In reality, it was far from simple and failed to provide clarity. If the person was intent on suicide and wanted to be left alone to get on with it, or was so detached from reality that they genuinely believed they were fine, they were likely to respond in the affirmative. This meant that the intervener still needed to exercise personal judgement, and quickly, as in the following example:

[c] The term embodied communication refers to instances in which one social actor conveys a message to another by means of the body or bodily activity alone (see Goffman E, *Behavior in Public Places*. New York; Free Press, 1963, p.13-14)

For peer review only - http://bmjopen.bmj.com/site/about/guidelines.xhtml

Rescued (1.06): "A woman turned up ... and she said, Are you OK? I said, I'm fine, thank you ... And she said, Well you don't seem fine. I said, I'm just looking at the view ... I was angry that she was meddling. It wasn't her business. I was perfectly fine on my own. She said, Mmm, do you want to climb back over and we can both look at the view? And I said, No."

Opening requests for information took other forms, such as asking what the person was doing there, which again did not always elicit a straightforward answer. In situations that were less ambiguous, interveners could begin with an assertion and/or an offer, as in the following example:

Intervener (2.10): "I approached very, very carefully. I didn't want to alarm or frighten her. I just said, Hello, my name's [X], I live in the flat up there. I can see you might be in a spot of bother. Is there anything I can do?"

Others were still more assertive, using verbal as well as physical restraint:

Rescued (1.07): "A woman came over ... She put her hand on my arm ... and she said, You don't need to do this. It's OK, nothing's that bad ... It sort of snapped me out of it for long enough that there was time for like, once you hesitate it's quite hard to go back from that."

Having approached the person and made an opening gambit, interveners then found themselves committed to remaining present for as long as it took, either to accomplish a handover to emergency services or to be sure that the person was no longer a risk to

For peer review only - http://bmjopen.bmj.com/site/about/guidelines.xhtml

themselves. In some cases this took several hours. During that time, they largely improvised, trusting their instincts, drawing on their personal resources, and trying to show warmth and compassion in whatever way they could. The following elements emerged as important in the accounts of both groups.

Exchanging names: Names were not always exchanged, but when they were this was seen as having played an important part in 'bursting the bubble' and personalising the encounter. By giving their name, interveners demonstrated that they were authentic or "real", which could have an anchoring effect:

> Rescued (1.06): "I remember her name. It's the one thing that I kept saying to myself … It was something to hold onto, more tangibly than like a physical thing, I think."

Asking simple, factual questions: Interveners went on to ask simple questions in the usual manner of 'getting to know you', enquiring where the person lived, whether they worked or were studying, and so on. This served multiple purposes, beyond establishing the facts. It gave the suicidal person something to focus on, drawing their attention away from imminent death and helping them to reconnect with themselves and their normal world. It also served gradually to build rapport between suicidal person and intervener. One participant highlighted the importance of persisting, if at first the suicidal person is unable to answer:

> Rescued (1.01): "It did take quite a lot of asking … She kept repeating the same questions … When she asked them the first time I wasn't in a place where I could answer, but as I sort of came back into reality a bit, rather than the world inside my

For peer review only - http://bmjopen.bmj.com/site/about/guidelines.xhtml

head … You start to feel a bit less disconnected … almost like it's foggy and then the fog lifts a bit, enough for you to see that there is something beyond the fog, like if the fog is suicide.”

Interveners also enquired about family members and friends, and if there was someone who could be contacted (see below, *Summoning help)*.

Sharing lived experience and personal stories: As in a normal conversation, interveners reciprocated by sharing information about themselves. When interveners had their own lived experience of mental health problems, participants in both groups believed it was useful to share this in order to establish credibility and trust and to provide hope of recovery. At the same time, interveners were careful to avoid focusing attention on themselves or presuming to know what the suicidal person was going through.

“Keeping it light”, and not asking why: Participants in both groups expressed a strong belief that this was not the time or place to probe the reasons for wanting to die. Those in the rescued group who had been asked what had brought them to the point of suicide reported consistently that they had been unable to talk about it, whilst others said they were glad they had not been asked. As they emerged from the ‘bubble’, their predominant feelings were those of ambivalence about being prevented from carrying out their plan, embarrassment and exhaustion, and their immediate need was simply for the comforting presence of another human being, not an intellectually demanding ‘talking therapy’ session that kept them focused on the distress:

For peer review only - http://bmjopen.bmj.com/site/about/guidelines.xhtml

Rescued (1.06): “She started saying, What’s happened? Why do you feel like this? And I didn’t say anything. I couldn't give her an answer. It was too complicated.”

Rescued (1.11): “She didn't ask why… I don't think I would have been able to answer her anyway. I would have probably bolted.”

Some interveners did ask what had brought the person to this point and some, but not all, received a response. Others reported that they had either shied away from “getting in too deep” or sensed that it was not appropriate to do so, believing that exploring the precipitating events or stressors could wait until later and that making the person dwell on their problems at this point would not be useful:

Intervener (2.13): “I realised I didn't need to go into the why, didn't need to establish any of that stuff.”

Nor was it considered helpful for a stranger to present a suicidal person with reasons for living. This was likely to be regarded as presumptuous and to provoke anger, especially when the intervener lacked lived experience of depression or suicidality.

Instead of dwelling on distress, interveners tried to keep the conversation “light” and focused on normal, everyday things. Whilst a number of them admitted that they were just trying to buy time or alleviate their own discomfort, rescued participants affirmed that this was nonetheless effective:

For peer review only - http://bmjopen.bmj.com/site/about/guidelines.xhtml

Intervener (2.13): "It was quite a random conversation … I don't like silence so I just filled it with things that popped into my head."

Rescued (1.05): "He just started rambling on about his own life, which was kind of lovely. Like he was just keeping me listening even if I wasn't talking … I could still relate to him in a way. He was saying he'd just come back from a party or something…"

Silences, and just being present: Others recognised that it was possible to connect with a person without words and that silence could provide the suicidal person with a welcome respite from the intrusive and negative babble that was going on inside their own head:

Rescued (1.03): "She didn't really talk too much. She was just there … And she stayed with me and bought me another cup of coffee … and eventually the voices were stopping and I felt like I was in the moment."

The mere presence of another person was also a powerful deterrent. Rescued participants consistently reported that the arrival of another person made it impossible to go through with their planned suicidal act. Even at the point of crisis, they still seemed able to think of others:

Rescued (1.08): "I wanted them to just go away, but they didn't … They were there and I didn't want to do anything in front of their little baby … because he was old enough to know what had happened."

For peer review only - http://bmjopen.bmj.com/site/about/guidelines.xhtml

Embodied communication or body language was as powerful as words in establishing a non-threatening presence and conveying warmth and empathy, and included the following elements.

Positioning of self: Interveners described how they positioned themselves vis-à-vis the suicidal person, placing themselves on an equal plane (e.g. sitting or squatting) and in the same orientation (alongside and facing the same way) so as to appear friendly rather than confrontational. Maintaining a respectful distance was seen as important by those who were suicidal:

> Rescued (1.09): "I remember saying, Don't come so close. I was really kind of adamant about that because this was my thing and my space."

Caring gestures and touch: Interveners attended to the suicidal person's physical needs, offering them a coat, a cigarette or a warm drink. The issue of touch was recognised by both groups as a difficult one. The warm touch of another human being could be very powerful in breaking the isolation of the suicidal person, but several in the rescued group reported that histories of abuse had left them with a horror of physical contact and so appreciated the intervener asking permission before touching:

> Rescued (1.03): "She said, Do you mind if I touch your hand? She asked me, and I can remember feeling the touch of a human person so clearly."

For peer review only - http://bmjopen.bmj.com/site/about/guidelines.xhtml

Rescued (1.06): “Her hand was on my arm and it was warm … I was this frozen ice person, who had given up on everything, and she'd come over with sort of warm words and this physically warm touch that I didn't necessarily want to pull away from.”

General demeanour: Those who had been rescued consistently reported that the manner in which the intervener spoke to them was as important as the words spoken, if not more so. Calmness (not showing alarm), authenticity (just being themselves, being “real”), steadfastness (“not budging”) and sincerity were traits that were mentioned again and again and that had the effect of making the person feel safe, valued and connected. The mere act of stopping and saying something gave a powerful signal to the person, at their lowest point, that they mattered:

Rescued (1.07): “The fact that you do stop makes all the difference, rather than walking past … The fact that [on three separate occasions] people stayed and talked to me … It was like, right now you matter and your life matters and we care what happens to you. And that was a genuine thing.”

ii) Moving to a safer location

Nearly all interveners had to work out how to move the person away from the means of suicide to a safer location. Efforts to do so took one of the following three forms.

Forcible restraint: Some interventions began with a physical act of ‘grabbing’, designed to effect the swift and forcible removal of a person from a situation of extreme danger, before any attempt was made to engage in verbal rescue work. Whilst no-one regarded

For peer review only - http://bmjopen.bmj.com/site/about/guidelines.xhtml

this as ideal, some rescued participants recognised that it had been the right thing to do in the circumstances:

> Rescued (1.01): "I remember hearing two people coming up the steps … and one of them just pulled me backwards off the ledge … Had I not been pulled down, I doubt I would have engaged at all because I was so inside of myself."

The risks involved in grabbing were also clearly illustrated. In one case, two women walking on a riverside path saw a person ahead of them on the wrong side of a bridge, leaning out over the water and in extreme peril. Having reached the person in time and grabbed both her arms, they then found themselves trapped, lacking the strength to pull her back over the railing, struggling to keep hold, and able neither to engage her in conversation nor to attract the attention of passing motorists. The situation was eventually resolved, but not without injury to one of the rescuers.

Grabbing was seen as a purely instrumental action, which, whilst it may have been effective in saving the life on that occasion, did nothing to contribute to longer-term recovery. Interventions at the other end of the control/agency spectrum (Figure 2), which encouraged self-rescue, were deemed more likely to have an ongoing positive and protective effect.

Direct appeal ("Please come away from the edge"): This was an option when there was less urgency and it was believed that the person had the capacity to respond. Some approached it in an oblique manner:

For peer review only - http://bmjopen.bmj.com/site/about/guidelines.xhtml

> Intervener (2.21):"I didn't want to confront her and say, Don't jump! … I said, Do you realise the cliff edge is really crumbly there? I'm quite worried that it might collapse. Why don't you step back a bit?"

Indirect ("Let's go somewhere for a coffee"): Offering to buy the person a warm drink and something to eat performed multiple purposes. It could be both a simple act of kindness and validation, and also a ploy to move the person to a safer location. Either way, it was a highly effective action, serving to ease the person back into the normal, everyday world (Figure 1):

> Intervener (2.12): "I was trying to work out how I could get her out of the tube and somewhere safer … So I said, Let's go and get a cup of tea somewhere and have some cookies … And she helped herself to a cookie and there were signs she was coming to a little bit … and she sort of opened up to me then."

iii) Summoning help

There were three potential sources of support on which interveners could call: a family member or friend nominated by the suicidal person; other passers-by, and the emergency services.

Family member or friend: Interveners typically asked about family and friends as part of 'bursting the bubble', but it was also a way to identify someone who might come and take the person home. The following example shows how a brief verbal exchange performed both tasks at once. The intervener appears initially to be trying to establish if there is someone who can be called to provide support. The suicidal person can think of no-one,

For peer review only - http://bmjopen.bmj.com/site/about/guidelines.xhtml

For peer review only

but as the exchange proceeds she gradually remembers her extended family and the closeness of her social bonds (Figure 1). The sight of the photograph on her phone represented a turning point, after which she could no longer go through with the act of suicide and agreed to contact one of her daughters:

> Rescued (1.03): "She said, Is there somebody you can ring? And I said, No, there's nobody. And she said, Have you got any children? And I said, Yes, I've got two daughters. Oh, have you got a picture, she said. And I got out my phone and showed her a picture of my six grandchildren…"

Other passers-by: Efforts to enlist help from other passers-by were rarely successful. Participants in both groups gave accounts of busy locations and people passing by, either not noticing or pretending not to notice what was happening. Several interveners made repeated and desperate attempts to signal to other passers-by but were ignored, although in some cases it transpired that a member of the passing crowd had taken it upon themselves to call emergency services. However, other interveners rejected offers of help, fearing that the presence of another person would threaten the one-to-one connection and trust they were establishing with the suicidal person.

Emergency services: Interventions typically ended with the arrival of emergency services. Rail staff and highways officers had usually alerted emergency services prior to approaching the suicidal person, so that their task was simply to keep the person safe until specialist help arrived. For members of the public it was much more challenging. None of them had thought to call emergency services prior to intervening, and once they had approached and embarked on rescue work it became very difficult to do so. They

For peer review only - http://bmjopen.bmj.com/site/about/guidelines.xhtml

feared that any move to do so, in particular any mention of police involvement, would trigger panic and flight. In some cases, the intervener asked permission to call for help and it was refused, leaving them alone and floundering. Others resorted to subterfuge or were fortunate that a member of the crowd called for help on their behalf, often unbeknown to them. Several interveners reported being unsure which service to ask for. The following example illustrates the multi-tasking nature of intervention work, the fragility of the connection with the suicidal person, and the impossibility of maintaining it whilst doing other things:

> Intervener (2.12): "I was trying to get my phone out of my handbag without losing eye contact … And I glanced down to unlock it and she ran … In that split second I lost her … So I was going after her and I was calling 999 as well, and I remember on the phone feeling quite confused because I really wasn't sure what service to be asking for ... I said, There's a lady running into the road, and they said, Well, what service do you need? And I said, I don't know..."

Interveners who had worked hard to build an emotional connection with the person expressed misgivings about medicalising the situation by calling an ambulance or criminalising it by involving the police.

Endings and aftermath

Ending an intervention without a handover to emergency services or to a family member or friend was fraught with difficulty. Interveners described being desperate to extricate themselves, sometimes after several hours, but not knowing whether it was safe to let the

For peer review only - http://bmjopen.bmj.com/site/about/guidelines.xhtml

person to go on their way and sometimes debating whether they should invite the person home with them.

Endings involving a handover were not necessarily any easier. Interveners experienced intense relief as they themselves were 'rescued', but were often left with feelings of exclusion, loss and fear of consequences for the person (especially when they were taken away in handcuffs or sectioned under the Mental Health Act). Some were left feeling disturbed by what had happened. Those who had been on their way to work at the time described the surrealness of trying to carry on *as normal* after the extraordinariness and intensity of the encounter.

For peer review only - http://bmjopen.bmj.com/site/about/guidelines.xhtml

DISCUSSION

This is the first empirical study to examine the role of passing strangers in preventing suicides. It demonstrates that anyone can prevent a suicide in a public place, and provides clear evidence-based principles to guide potential interveners.

Suicidal people typically described themselves as being dissociated or "in a bubble," cut off from themselves and everything around them. Dissociative states have long been linked with suicide.[19] Orbach postulates that detachment from the body, indifference to physical pain and lack of affect are necessary in order for an individual to go through with a suicidal act. Absence of emotion is a subtle and counterintuitive clue. In this regard, suicide is like drowning insofar as the person does not look like we expect them to. A drowning person is not thrashing about and shouting for help, as commonly portrayed in film; they appear silent and still and are easily overlooked. Likewise the suicidal person rarely conforms to the stereotypical image of the anguished soul, as depicted in Munch's painting of The Scream. This is an important message to convey to the public. In an online survey of rail staff, not all of whom had direct experience of dealing with suicides on the railways, Mackenzie and colleagues found that a high proportion of staff expected suicidal individuals to exhibit visible signs of distress.[20]

With few outward clues except location, interveners relied on their own 'sixth sense' that something was wrong. Some quality of oddness or incongruity triggered an alarm inside them, which they were able to hear. Thus recognition relies as much on an *internal* state of openness, curiosity, attunement to others and attention to one's own inner voice as on any *external* signs.

For peer review only

Openness needs to be combined with readiness for action. The interveners in our study were ordinary people; they did not possess any special knowledge or skills. What is unusual about them is their lack of hesitancy and their disregard of the social risks involved in approaching a stranger in a public place. Most people are kept from doing so by fear of a rude rebuff and resulting embarrassment.[21] The interveners we studied were not fearless but, on the principle of “Feel the fear and do it anyway”, they did it anyway. They also showed themselves to be resourceful, able to dig around in their personal ‘experience bag’ and find things that worked, without necessarily knowing how or why.

Our findings suggest that people do not need to be provided with an intervention script, nor should they be afraid of saying ‘the wrong thing.’ The words the intervener spoke were not nearly as important as how they made the person feel. The rescued group described interveners as having broken through the dissociative state, dispelling feelings of fear, isolation and worthlessness and making them feel safe, validated and re-connected. The basic mechanisms are depicted in Figure 3.

[INSERT FIGURE 3]

This model of effective intervention, with feeling rather than reasoning at its core, runs counter to conventional wisdom, as contained in programmes such as Applied Suicide Intervention Skills Training (ASIST)[22] and in academic literature.[8] The ASIST model stresses the importance of listening closely to the person’s reasons for dying and working with them to uncover reasons for living. Its focus is on verbal reasoning. Likewise, Omer and Elitzur’s proposed script for a ‘Stranger on the Bridge’ intervention, based on clinical

For peer review only - http://bmjopen.bmj.com/site/about/guidelines.xhtml

practice, insists on challenging suicidal thinking. They compare the situation to a court of law in which the defence lawyer (the intervener) picks holes in the prosecutor's (the suicidal person's) story in an attempt to win the argument.[8] Our interveners were not attempting to win a legal battle. Nor were they trying to imitate therapists or crisis negotiators. They were just being themselves and reaching out as one human being to another. This is not to say that reasoning and verbal persuasion played no part, or that they may not be important at other stages of the suicidal process, but few of our suicidal individuals reported having been swayed by logic or argument. What they recalled most strongly about those who had helped them was their authenticity and their compassion. What made the difference was the simple fact that someone cared enough not to walk on by.

Our model may not be appropriate to other settings. The 'Stranger on the Bridge' scenario, involving an unknown person on the point of carrying out a suicide plan in a public location, is very different from other crisis settings. These include domestic or social settings, where a personal relationship exists between the two parties;[15] clinical settings, where a professional relationship and statutory responsibility for risk management exist,[23-25] and telephone counselling services, where the suicidal person has invited a talking intervention by calling a crisis line.[26, 27] In these cases, the person may not yet be at the stage having formulated a suicide plan. Our study suggests that rather than there being a 'one size fits all' model of human intervention, as the ASIST training supposes, different social contexts and stages of the suicidal process may require different approaches.

For peer review only - http://bmjopen.bmj.com/site/about/guidelines.xhtml

Our findings show clearly that anyone who wants to intervene can safely do so. Last-minute intervention requires no specific learning and can be highly effective when spontaneous and unscripted. In 2018, Network Rail in partnership with Samaritans launched a campaign entitled "Small Talk Saves Lives", encouraging rail travellers simply to say 'Hello' and strike up a normal conversation if they are concerned about someone.[28] Our data confirm that this campaign message is entirely appropriate as far the initial approach to a vulnerable person is concerned. However, it fails to recognise the intense, prolonged and taxing nature of intervention, the complex juggling acts that interveners may have to perform in the course of trying to keep someone safe, and the troubling emotions they may be left with. It is no small thing to save a life. The conundrum for public education is how to prepare people adequately for the challenges they may face without deterring them from intervening.

A set of simple public education messages is needed to empower people to recognise and reach out to those who may be at risk of suicide in public locations. We are working with a wide range of non-academic partners to develop and disseminate these.

For peer review only - http://bmjopen.bmj.com/site/about/guidelines.xhtml

ACKNOWLEDGEMENTS

We thank the participants in both groups for sharing their stories. We are also grateful to Network Rail and Highways England for help with recruiting interveners, to Jonny Benjamin MBE, Neil Laybourn (the original 'Stranger on the Bridge'), and the other members of our expert advisory group.

FUNDING STATEMENT

This work was supported by the Medical Research Council grant number MR/P01707X/1.

COMPETING INTERESTS

The authors declare that they have no competing interests.

AUTHOR CONTRIBUTIONS

CO had the idea for the study and conducted public involvement. She designed the study with CA, managed the project, led the analysis and interpretation of data and wrote the manuscript. JD collected data and analysed the transcripts. Both JD and CA commented on emerging drafts of the manuscript. All authors have read and approved the final version. CO is the guarantor.

For peer review only

REFERENCES

1. Owens C, Lloyd-Tomlins S, Emmens T, et al. Suicides in public places: findings from one English county. *European Journal of Public Health* 2009;19(6):580-582.
2. Channel 4: http://www.channel4.com/programmes/the-stranger-on-the-bridge 2015 [Accessed 07.06.2019].
3. Department of Health. *Preventing suicide in England: A cross-government outcomes strategy to save lives*. London: HM Government, 2012
4. Owens C, Hardwick R, Charles N, et al. *Preventing suicides in public places: A practice resource*. London: Public Health England, 2015. https://www.gov.uk/government/publications/suicide-prevention-suicides-in-public-places.
5. Cox G, Owens C, Robinson J, et al. Interventions to reduce suicides at suicide hotspots: A systematic review. *BMC Public Health* 2013;9(13):214.
6. Pirkis J, Spittal M, Cox G, et al. The effectiveness of structural interventions at suicide hotspots: a meta-analysis. *Int J Epidemiol* 2013;42(2):541-548.
7. Pirkis J, Too L, Spittal M, et al. Interventions to reduce suicides at suicide hotspots: a systematic review and meta-analysis. *The Lancet Psychiatry* 2015;2(11):994-1001.
8. Omer H, Elitzur A. What would you say to the person on the roof? A suicide prevention text. *Suicide Life Threat Behav* 2001;31(2):129-139.
9. World Health Organization. *Preventing Suicide: A Resource for Police, Firefighters and Other First Line Responders.* Geneva: World Health Organization, 2009. http://www.who.int/mental_health/prevention/suicide/resource_firstresponders.pdf [Accessed 07.06.2019]
10. Lanceley F. *On-Scene Guide for Crisis Negotiators (2nd edition)*. Boca Raton, Fl: CRC Press, 2003.

For peer review only - http://bmjopen.bmj.com/site/about/guidelines.xhtml

11. Greenstone J. Teen Suicide: Negotiating with the Young Suicidal Actor. *Journal of Police Crisis Negotiations* 2008;3(2):61-64.

12. Fowler W, Maguire J. Dealing with Defenestrators: Immediate Interventions. *Journal of Police Crisis Negotiations* 2001;1(2):41-51.

13. Holloway W, Jefferson T. *Doing Qualitative Research Differently: Free Association, Narrative and the Interview Method*. London: Sage, 2000.

14. Wengraf T. *Qualitative Research Interviewing: Biographic Narrative and Semi-Structured Methods.* London: Sage, 2001.

15. Owens C, Owen G, Belam J, et al. Recognising and responding to a suicidal crisis in the family and social network: qualitative study. *BMJ* 2011;343:d5801.

16. Cannon S. Social research in stressful settings: difficulties for the sociologist studying the treatment of breast cancer. *Sociol Health Illn* 1989;11(1):62-77.

17. Lee RM. *Doing research on sensitive topics*. London: Sage, 1993.

18. Holstein K, Gubrium J. Active interviewing. In: Silverman D, ed. *Qualitative Research: Theory, Method and Practice.* London: Sage, 1997.

19. Orbach I. Dissociation, physical pain and suicide: A hypothesis. *Suicide and Life-Threatening Behavior* 1994;24(1):68-79.

20. Mackenzie J, Borrill J, Hawkins E, et al. Behaviours preceding suicides at railway and underground locations: a multimethodological qualitative approach. *BMJ Open* 2018;8:e021076.

21. Goffman E. *Behavior in Public Places: Notes on the Social Organization of Gatherings.* New York: Free Press, 1963.

22. https://www.livingworks.net/programs/asist/

For peer review only - http://bmjopen.bmj.com/site/about/guidelines.xhtml

23. Appleby L, Morriss R, Gask L, et al. An educational intervention for front-line health professionals in the assessment and management of suicidal patients (The STORM Project). *Psychological Medicine* 2000;30(4):805-812.

24. Hendin H, Maltsberger J, Lipschitz A, et al Recognizing and responding to a suicide crisis. *Suicide Life Threat Behav* 2001;31(2):115-128.

25. Department of Health. *Best Practice in Managing Risk: Principles and Evidence for Best Practice in the Assessment and Management of Risk to Self and Others in Mental Health Services.* London: Department of Health, 2009. https://assets.publishing.service.gov.uk/government/uploads/system/uploads/attachment_data/file/478595/best-practice-managing-risk-cover-webtagged.pdf [Accessed 07.06.2019].

26. Mishara B, Chagnon F, Daigle M, et al. Which helper behaviors and intervention styles are related to better short-term outcomes in telephone crisis intervention? Results from a Silent Monitoring Study of Calls to the U.S. 1-800-SUICIDE Network. *Suicide Life Threat Behav* 2007;37(3):308-321.

27. Gould M, Cross W, Pisani A, et al. Impact of Applied Suicide Intervention Skills Training on the National Suicide Prevention Lifeline. *Suicide and Life Threatening Behavior* 2013;43(6):676-691.

28. https://www.samaritans.org/media-centre/our-campaigns/small-talk-saves-lives [Accessed 07.06.2019].

For peer review only - http://bmjopen.bmj.com/site/about/guidelines.xhtml

Figure 1: Main intervention tasks, components and interconnections

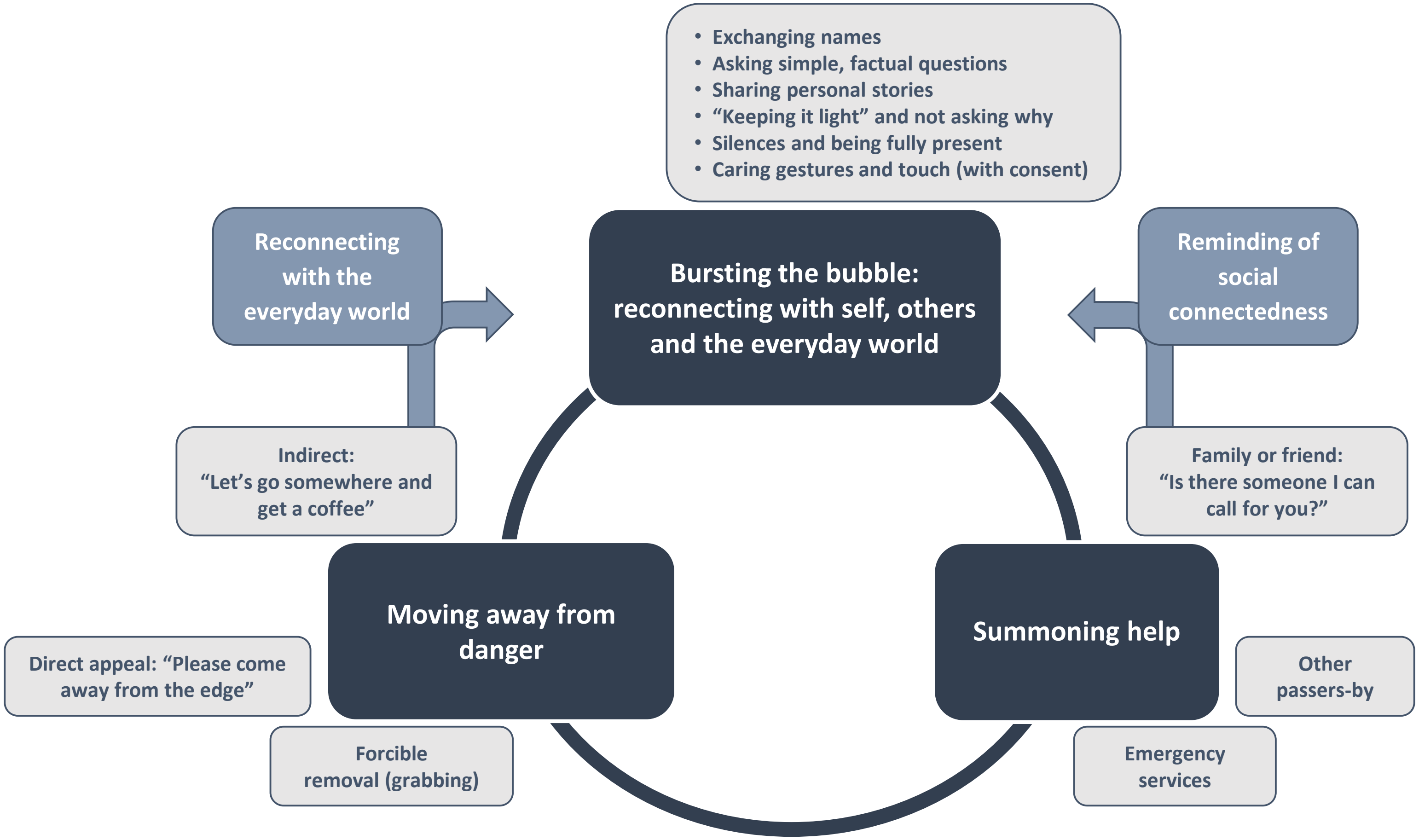

Figure 2: Control/agency spectrum

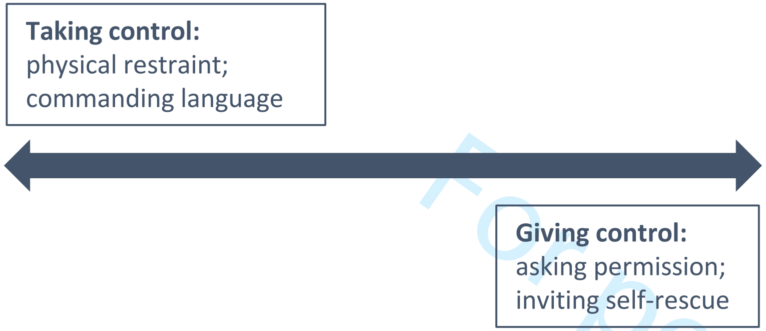

For peer review only

Figure 3: Proposed mechanisms of action in a 'Stranger on the Bridge' intervention

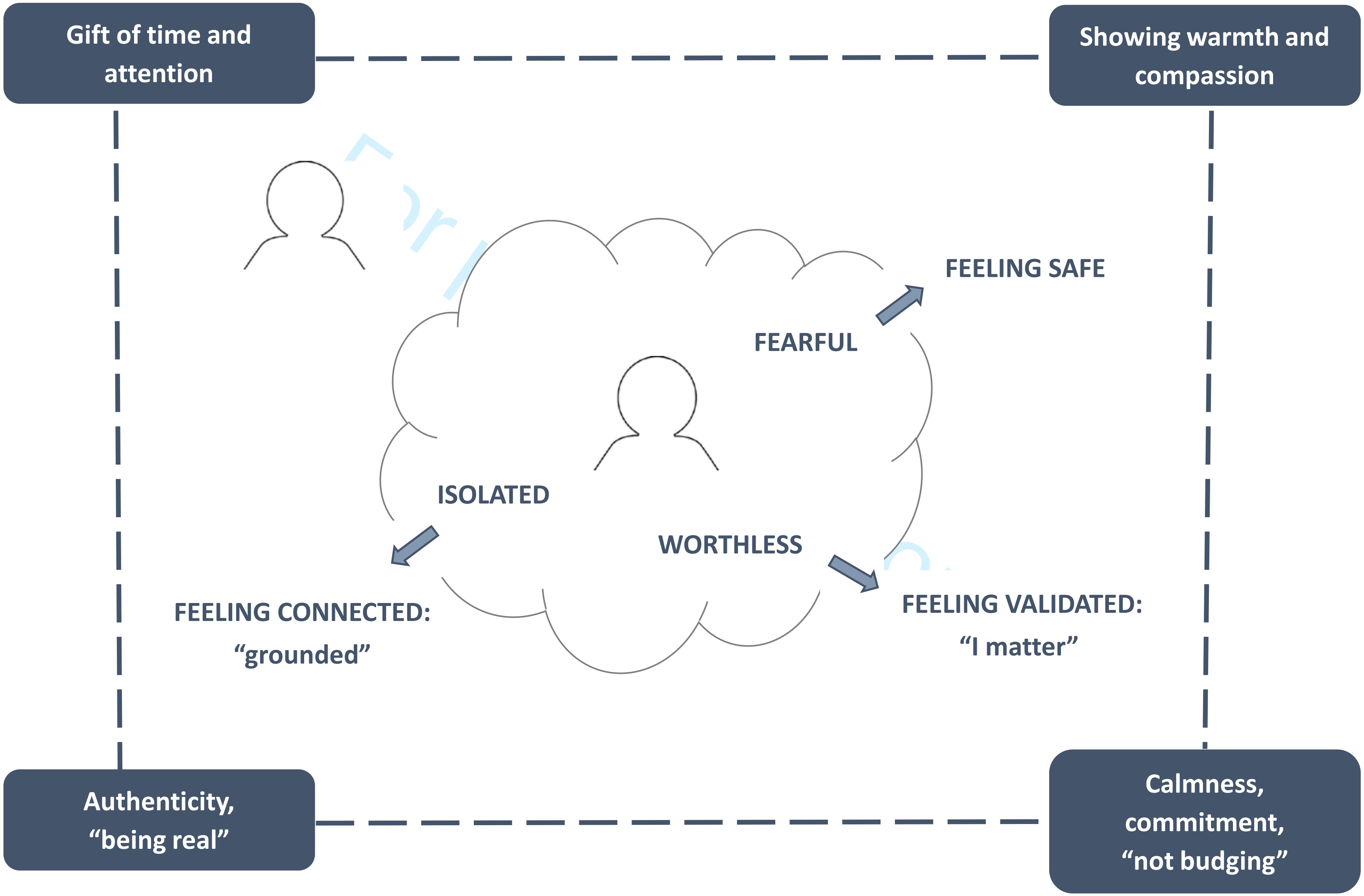

Consolidated criteria for reporting qualitative studies (COREQ): 32-item checklist

**Guide questions/description**	**Authors' response**
Domain 1: Research team and reflexivity	
Personal Characteristics<br>1. Interviewer/facilitator. Which author/s conducted the interview or focus group?	Reported in manuscript
2. Credentials. What were the researcher's credentials? E.g. PhD, MD	Master's degree and PhD
3. Occupation. What was their occupation at the time of the study?	University Research Fellow
4. Gender. Was the researcher male or female?	Female
5. Experience and training. What experience or training did the researcher have?	Extensive track record in use of qualitative research methods.
Relationship with participants<br>6. Relationship established. Was a relationship established prior to study commencement?	Email and telephone contact prior to interview
7. Participant knowledge of the interviewer. What did the participants know about the researcher? e.g. personal goals, reasons for doing the research	The interviewer shared relevant personal information during preliminary conversations.
8. Interviewer characteristics. What characteristics were reported about the interviewer/facilitator? e.g. Bias, assumptions, reasons and interests in the research topic	Insufficient space to report
Domain 2: study design	
Theoretical framework<br>9. Methodological orientation and Theory. What methodological orientation was stated to underpin the study? e.g. grounded theory, discourse analysis, ethnography, phenomenology, content analysis	Inductive thematic analysis
Participant selection<br>10. Sampling. How were participants selected? e.g. purposive, convenience, consecutive, snowball	There are a limited number of people in the general population who meet the inclusion criteria and they are impossible to identify except by advertising the study and hoping they will come forward. This was the only possible sampling strategy.
11. Method of approach. How were participants approached? e.g. face-to-face, telephone, mail, email	Reported in manuscript
12. Sample size. How many participants were in the study?	Reported in manuscript
13. Non-participation. How many people refused to participate or dropped out? Reasons?	n/a
Setting<br>14. Setting of data collection. Where was the data collected? e.g. home, clinic, workplace	Reported in manuscript
15. Presence of non-participants. Was anyone else present besides the participants and researchers?	No

For peer review only

16. Description of sample. What are the important characteristics of the sample? e.g. demographic data, date	Reported in manuscript
Data collection<br>17. Interview guide. Were questions, prompts, guides provided by the authors? Was it pilot tested?	Reported in manuscript
18. Repeat interviews. Were repeat interviews carried out? If yes, how many?	No repeat interviews
19. Audio/visual recording. Did the research use audio or visual recording to collect the data?	Reported in manuscript
20. Field notes. Were field notes made during and/or after the interview or focus group?	Yes
21. Duration. What was the duration of the interviews or focus group?	Reported in manuscript
22. Data saturation. Was data saturation discussed?	Reported in manuscript
23. Transcripts returned. Were transcripts returned to participants for comment and/or correction?	No. It was not considered ethical for the 'rescued' group to read accounts of their own past suicidal crises without a researcher present to offer support should they become distressed or find the accounts triggering.
Domain 3: analysis and findings	
Data analysis<br>24. Number of data coders. How many data coders coded the data?	One coded all transcripts using NVivo; one coded independently using non-computerised methods and also checked NVivo.
25. Description of the coding tree. Did authors provide a description of the coding tree?	Available from the authors
26. Derivation of themes. Were themes identified in advance or derived from the data?	Reported in manuscript
27. Software. What software, if applicable, was used to manage the data?	Reported in manuscript
28. Participant checking. Did participants provide feedback on the findings?	No, see above, 23. We presented findings on three occasions to an expert advisory group comprising people with lived experience and a range of external stakeholders.
Reporting<br>29. Quotations presented. Were participant quotations presented to illustrate the themes / findings? Was each quotation identified? e.g. participant number	Yes
30. Data and findings consistent. Was there consistency between the data presented and the findings?	Yes
31. Clarity of major themes. Were major themes clearly presented in the findings?	Yes
32. Clarity of minor themes. Is there a description of diverse cases or discussion of minor themes?	Yes

For peer review only

BMJ Open

Intervening to prevent a suicide in a public place: A qualitative study of effective interventions by lay people

Journal:	*BMJ Open*
Manuscript ID	bmjopen-2019-032319.R1
Article Type:	Original research
Date Submitted by the Author:	03-Sep-2019
Complete List of Authors:	Owens, Christabel ; University of Exeter, College of Medicine and Health<br>Derges, Jane; University of Exeter, College of Medicine and Health<br>Abraham, Charles; University of Melbourne, School of Psychological Sciences
**Primary Subject Heading**:	Public health
Secondary Subject Heading:	Mental health, Qualitative research
Keywords:	Suicide & self-harm < PSYCHIATRY, PUBLIC HEALTH, QUALITATIVE RESEARCH, MENTAL HEALTH

SCHOLARONE™
Manuscripts

Intervening to prevent a suicide in a public place:

A qualitative study of effective interventions by lay people

Authors:

Christabel Owens,* University of Exeter Medical School, Exeter, UK

Jane Derges, University of Exeter Medical School, Exeter, UK

Charles Abraham, School of Psychological Sciences, University of Melbourne, Australia

*** Corresponding author:**

Professor Christabel Owens, University of Exeter Medical School, College House,

St Luke's Campus, Exeter EX1 2LU, United Kingdom.

Tel: +44 (0)1392 726006

Email: c.v.owens@exeter.ac.uk

For peer review only

For peer review only - http://bmjopen.bmj.com/site/about/guidelines.xhtml

ABSTRACT

Objectives: Many suicides take place in public locations, usually involving jumping from high places or use of transport networks. Previous research has largely focused on the effectiveness of physical barriers at frequently-used locations. There have been no studies of human intervention to prevent suicides in public places. The aim of this study was to identify the core components of an effective intervention by a member of the public.

Design and methods: We conducted in-depth qualitative interviews with people who have either been prevented by a stranger from taking their own life in a public location (n=12), or intervened to prevent a stranger from taking their own life in a public location (n=21). Collectively, the two groups narrated 50 incidents of suicide rescue. We analysed interview transcripts using inductive thematic analysis.

Results: Suicidal people typically displayed no visible distress, describing themselves as being dissociated or "in a bubble." Intervention was seen to involve three main tasks: "Bursting the bubble" (reconnecting with self, others and everyday world); Moving to a safer location, and Summoning help. We show how interveners accomplished these tasks in a range of ways, using both verbal and non-verbal communication and different degrees of restraint.

Conclusions:

This is the first empirical study to examine the role of passing strangers in preventing suicides in public places. It shows that no specialist skills are needed. Interveners were ordinary people, distinguished only by a high level of social awareness, combined with a

For peer review only

For peer review only - http://bmjopen.bmj.com/site/about/guidelines.xhtml

readiness for social action. The findings also suggest that people do not need a script and should not be afraid of saying "the wrong thing." What interveners said was much less important than how they made the suicidal person feel, namely safe, connected and validated ("I matter"). Interveners did this simply by being themselves, responding with authenticity, calmness and compassion. Members of the public need to be encouraged to recognise and reach out to those who may be at risk of suicide in public locations, but should be prepared for a prolonged and intense encounter that may leave them with troubling emotions.

For peer review only

STRENGTHS AND LIMITATIONS OF THIS STUDY

- This is the first empirical study to examine the role of passing strangers in preventing suicides.
- We included the perspectives of both interveners and those whose lives had been saved by the intervention of a stranger and found a very high level of agreement between them as to what worked, giving confidence in our conclusions about effective interventions.
- Although we did not specify that interventions must have been successful, our intervener group did not include any individuals who had failed to avert a suicide, so we are unable to draw any conclusions about ineffective interventions.
- We allowed a wide range in the length of time between incident and interview (from less than one year to more than ten years), extending our original upper limit of five years in some cases, after careful screening to assess the quality of recall; all participants were able to give extremely detailed accounts, which we had no reason to doubt.
- Our model of effective suicide crisis intervention may be culturally-specific and may not be suited to other countries, particularly those in east Asia that have much higher incidence of suicides in public places.

For peer review only

INTRODUCTION

Suicide accounts for around 6,000 UK deaths each year, up to a third of which occur in public locations.[1] These may be more easily preventable than those that occur in the privacy of the home, because of the potential for a chance passer-by to make a last-minute intervention. There is compelling anecdotal evidence that lives can be saved by passing strangers acting on the spur of the moment. *The Stranger on the Bridge* was an award-winning documentary, broadcast on UK television in 2015, which told the story of one such intervention and captured global media attention.[2]

Preventing suicides in public places occupies a key place in England's national suicide prevention strategy.[3] In 2015 Public Health England published guidance for local authorities to help them identify sites and structures that might be used for suicides and take action to prevent deaths at those locations.[4] These typically include sites that provide opportunity for jumping from a height (bridges, high buildings and cliffs) and sections of the road and railway networks. Previous research has largely focused on the effectiveness of installing physical barriers at such locations to restrict access to means.[5-7] There are no studies of human intervention, and no evidence-based guidance for members of the public. The literature contains only one published article on the topic of what a potential helper might say in a last-minute crisis intervention.[8] Based on the authors' clinical experience rather than research evidence, it provides a suggested script that is long, verbose and contains no clear messages for the general public; nor has it been tested. The World Health Organization (WHO) has published guidance for emergency services personnel responding to suicidal crises,[9] but the advice is very general and does not differentiate between crises occurring in private and in public places. There is an extensive literature on police crisis negotiation, some of which deals with suicide

For peer review only - http://bmjopen.bmj.com/site/about/guidelines.xhtml

intervention,[10-12] but this is either too general, in the manner of the WHO document, or too specialized, describing 'tricks of the trade' that could not be safely recommended to lay people.

We were not interested in the part played by health professionals or members of the emergency services, whose jobs include suicide crisis intervention, but in ordinary citizens who happen to be passing by. The aim of this study was to identify the core components of an effective intervention by a stranger in a public place.

For peer review only - http://bmjopen.bmj.com/site/about/guidelines.xhtml

METHODS

Design

We conducted a qualitative interview study in order to understand intervention by a passing stranger from the perspective of both parties.

Participants and sampling

Participants were eligible for inclusion if they were aged 18 or over and had experience of either of the following:

- Being stopped from taking their own life in a public location by a passing stranger (Survivors);
- Trying to stop someone they did not know from taking their own life in a public location (Interveners). This group included members of the general public and members of staff in non-health agencies whose work at public locations brings them into contact with suicidal individuals, such as railway staff.

A public location was defined as anywhere outside their own or someone else's home. For both groups, we placed advertisements on the websites of mental health and suicide prevention charities and used the social media accounts (Facebook and Twitter) of non-academic partners to invite people with relevant experience to come forward. Interveners were also sought via relevant agencies, including Network Rail, Highways England and bridge authorities. We continued recruiting until each group contained a reasonable degree of diversity in respect of age, gender and ethnicity and we were confident that no new insights were emerging (data saturation).

Interviews

For peer review only - http://bmjopen.bmj.com/site/about/guidelines.xhtml

We conducted in-depth interviews with both groups, beginning with an invitation to give an uninterrupted account: “Please tell me in your own words as much as you can remember about [the event in question].” This free narrative approach affords insight into what is uppermost in the participant’s mind.[13-15] It also allows them to disclose information at their own pace, thereby increasing their sense of safety when talking about difficult or sensitive subjects.[16 17] During the narrative phase the interviewer [JD] assumed the role of active listener. She then proceeded to ask open-ended questions, following up any points requiring clarification and probing for further detail where necessary.[18] Separate interview guides for each group were used to ensure that all topics of interest were covered and that safety protocols were followed [available as supplementary files]. Interveners reflected in detail on what they said/did and how key decisions were made. Survivors described what the intervener said/did and how it affected them, allowing us to examine mechanisms of action.

All interviews with survivors were conducted face-to-face, mostly in participants’ homes, and lasted 1-2 hours. Interviews with interveners were conducted face-to-face (n=15), by telephone (n=4) or Skype (n=2). All participants gave written consent. Interviews were audio-recorded, transcribed verbatim and anonymised.

Analysis

Data collection and analysis were carried out concurrently to allow for progressive focusing of interviews and exploration of emerging ideas. Two investigators [CO and JD] independently read the first three transcripts from each group (n=6) before meeting to discuss candidate themes and analytic strategy. They then worked line-by-line through six further transcripts, noting and naming specific features of the situation, including:

For peer review only - http://bmjopen.bmj.com/site/about/guidelines.xhtml

characteristics of the actors; discrete intervention elements (verbal and non-verbal) and their effects on the suicidal person; constraints, complicating factors and tipping points. These were used to construct a list of categories, with definitions, that were used for coding the entire data set. Coding, sorting and retrieval were carried out using NVivo software. We then carefully examined all the coded material, exploring each category and its relationship to others, visually mapping intervention elements, and working via a series of iterations and data checks towards our final interpretive model.

Patient and public involvement

We formulated the question and designed the study in consultation with people with relevant lived experience, representatives from suicide prevention charities and the transport industry. They were instrumental in recruiting participants and subsequently commenting on findings and interpretation. We will continue to work with them as we start to frame key public education messages. A lay summary of results was sent to participants, who were also invited to help with future message development work.

For peer review only

RESULTS

Characteristics of the sample

We recruited 12 people who had been stopped from taking their own life in a public place (Survivors): nine female and three male, with ages ranging from 19-64. Four of them had been stopped on more than one occasion (one twice and three on three occasions), giving a total of 19 accounts of successful intervention.

The Intervener group contained 21 individuals: nine male and twelve female, also ranging in age from 19-64. This number was made up of 13 members of the general public, six railway workers[a] (two of whom were off-duty at the time) and two highways officers. Between them they had conducted 31 interventions, all successful. Most of the railway workers and one of the highways officers had intervened successfully on more than one occasion. Three members of the public had conducted more than one rescue. One participant was included in both groups because, having been stopped from taking her own life in a public place, she went on to conduct two successful interventions. The members of the public included students, teachers/lecturers, youth leaders, church and charity workers, civil servants, office workers and an actor. Several of them had lived experience of mental health problems. They recounted how, during the intervention, they drew on a miscellany of skills and scraps of knowledge that they had acquired in other spheres, including work and leisure pursuits. One used the metaphor of an 'experience

[a] The railway staff were all Mobile Operations Managers, whose role is to deal with a range of incidents on the railway network, including threatened suicides and fatalities. They differed from members of the general public insofar as: a) they were often acting on a referral, so had some time to prepare; b) five out of the six had received basic suicide intervention training from Samaritans; c) they had responsibilities beyond caring for the suicidal person, including restoring train services as quickly as possible, and d) they had organisational arrangements in place for managing such incidents, including direct links to the British Transport Police. Our rationale for including them in the intervener group was that they were responding as 'lay people', i.e. not as mental health professionals, to a threat of suicide by a stranger in a public location.

For peer review only - http://bmjopen.bmj.com/site/about/guidelines.xhtml

bag' that she carried around with her and had raided for ideas and possible strategies to use while struggling on two different occasions to keep a suicidal person safe.

Of the 50 interventions recounted (19 by survivors and 31 by interveners), 27 occurred on the rail and road networks (including bridges over road or railway). A further 18 involved other types of bridge or high places. Characteristics of participants and settings are shown in Table 1.

Table 1: Participants and settings

	Survivor group (n=12)	Intervener group (n=21)
Age range	19-64	19-64
Gender:		
Female	9	12
Male	3	9
Ethnicity:		
White British/European	10	21
Asian/Asian British	2	-
Black/Black British	-	-
Other ethnic groups	-	-
Mixed/Multiple ethnicity	-	-
Location:	(n of incidents =19)	(n of incidents =31)
Rail/underground/bridge over railway	4	14
Road network/bridge over road	2	8
River/bridge over river	7	4
High building	3	0
Cliffs	2	3
Other	1	2
Time since incident:	(n=19)	(n=31)
1 year or less	7	7
2 – 5 years	8	10
6 - 10 years	2	12
More than 10 years	2	2

We present our findings under the following headings: Recognition; Three intervention tasks ("Bursting the bubble," Moving to a safer location, and Summoning help), and Endings and aftermath.

For peer review only - http://bmjopen.bmj.com/site/about/guidelines.xhtml

Recognition

The prerequisite for intervention is recognition. Interveners recognised the person as being at risk or in need of help primarily by their location. The person either appeared to have placed themselves in immediate physical danger or was judged to be out of place, giving rise to curiosity or concern:

> *Intervener (2.06):"There was a person standing there in a hoodie, sort of facing the railway and looking over the side … and I took a couple of steps and thought, Hang on, what's she doing there? I did literally stop and think, Hang on, this is odd."*

Survivors also acknowledged the oddness of their positioning:

> *Survivor (1.01):"I went very close to the edge of the platform and sat with my feet over the edge … quite hunched up, possibly my hands over my ears and my eyes closed. I mean, it's not generally a position people take when they're waiting for a train."*

There were few clear behavioural clues and rarely anything in their mode of dress or appearance to mark them out. What was striking in interveners' accounts of what they saw was the absence of visible emotion. They described the suicidal person as looking "vacant", "glazed over", "zoned out" or "as if no-one was at home", but not distressed.[b] This was corroborated in the accounts of survivors, who described themselves as having

[b] There were two instances recounted by interveners in which the person was sobbing inconsolably. In both cases the person spoke of wanting to end their life, but neither was in a high-risk location or in possession of a means of suicide, suggesting that they were not about to carry out a plan. This was confirmed by a survivor (1.01), who said, *"I wouldn't even be able to walk to the station if I was crying that much. It would be when I've run out of tears … and I just feel empty and numb and disconnected. That's when I'm much more likely to act."*

For peer review only - http://bmjopen.bmj.com/site/about/guidelines.xhtml

moved into a space beyond emotion: "a weird sort of surreal, unfeeling state" in which they were "completely numb", "frozen" or "dissociated", cut off from themselves, others and the everyday world, to the point where they believed they were invisible. They were not inviting rescue:

Survivor (1.11): "It didn't occur to me that people would stop and take notice. I was very much of this mindset that I was invisible and no-one would see me."

This state of dissociation was described as being "in a bubble".

Interveners, on the other hand, appear to have been characterised by a state of openness to what was going on around them and an attunement to human distress even when it was not manifest. Some reflected that this had been acquired from parents with a strong sense of social justice or through experience in particular settings, such as working as a bar manager: "They teach you to spot trouble before it happens" (Intervener 2.15). This participant had been walking across a bridge with his partner when they encountered a person preparing to take her own life. He knew immediately that something was amiss, whilst his partner was unaware of anything until he started to intervene.

Three intervention tasks

We identified three main tasks involved in intervention:

i) "Bursting the bubble" (reconnecting with self, others and the everyday world);

ii) Moving to a safer location;

iii) Summoning help.

For peer review only - http://bmjopen.bmj.com/site/about/guidelines.xhtml

The three tasks are interconnected, as shown in Figure 1 and described below. Interventions were typically complex and interveners' accounts, particularly those of general public members who did not have organisational structures around them, show them to be frantically multi-tasking during the course of the encounter, trying to think ahead and plan several possible moves like chess players. Occasionally they managed to achieve several purposes through a single speech act or gesture.

[INSERT FIGURE 1]

The order in which they approached the tasks and the manner of intervention was influenced by a number of factors, including: the immediacy of danger; clarity of suicidal intent; the capacity of the suicidal person to interact verbally; the intervener's personality, and whether they were in a position of authority and responsibility. Interventions and their constituent parts fell on a spectrum according to the degree of control assumed by the intervener (Figure 2). At one end were interventions involving forcible restraint and authoritative language, often necessitated by the perceived urgency of the situation. At the opposite end, some interveners were acutely mindful of the suicidal person's right to self-determination and need to feel in control of their own destiny. Accordingly, they asked permission before taking any action and used invitations and appeals, calling on the person to rescue themselves and allowing them to keep all their options open:

Survivor (1.05): "He came up behind and he said, Look, I don't want to stop you from doing anything but I'd like it if you could come over the barrier … Just step on over, it's OK … He said, I'm not going to take you away from here, you can stay here if you want …

For peer review only

It was like, he's not forcing me to do anything. I can still do it ... and that was important for me."

[INSERT FIGURE 2]

i) "Bursting the bubble"

Both interveners and survivors used the metaphor of the bubble and described intervention in terms of bringing the suicidal person out of their trance-like, dissociated state and back into the present moment, reconnecting them with themselves, others and the everyday world. This emotional labour formed the heart of most of the interventions we studied. It was predominantly verbal in nature, although embodied communications sometimes also played a key part.[c]

Initial approach: The work began with the intervener establishing their presence. Most approached cautiously and started with some kind of question, such as:

"Are you OK?"

"Can I ask what you're doing there?"

"You seem to be in a spot of trouble. Can I help?"

These apparently simple enquiries did not always elicit a straightforward answer. The intervener still needed to exercise personal judgement, and quickly, as in the following example:

[c] The term embodied communication refers to instances in which one social actor conveys a message to another by means of the body or bodily activity alone (see Goffman E, *Behavior in Public Places*. New York; Free Press, 1963, p.13-14)

For peer review only - http://bmjopen.bmj.com/site/about/guidelines.xhtml

Survivor (1.06): "A woman turned up … and she said, Are you OK? I said, I'm fine, thank you … And she said, Well you don't seem fine. I said, I'm just looking at the view. I was angry that she was meddling. It wasn't her business. I was perfectly fine on my own. She said, Mmm, do you want to climb back over and we can both look at the view? And I said, No."

Other interveners were more assertive in their approach, using some degree of verbal or physical restraint:

Survivor (1.07): "A woman came over … She put her hand on my arm … and she said, You don't need to do this. It's OK, nothing's that bad."

Having approached the person and made an opening gambit, interveners then found themselves committed to remaining present for as long as it took, either to accomplish a handover to emergency services or to be sure that the person was no longer a risk to themselves. In some cases this took several hours. During that time, they largely improvised, trusting their instincts, drawing on their personal resources, and trying to show warmth and compassion in whatever way they could. The following elements emerged as important in the accounts of both groups.

Exchanging names: Names were not always exchanged, but when they were this was seen as having played an important part in "bursting the bubble" and personalising the encounter. By giving their name, interveners demonstrated that they were authentic or "real", which could have an anchoring effect:

For peer review only - http://bmjopen.bmj.com/site/about/guidelines.xhtml

Survivor (1.06): "I remember her name. It's the one thing that I kept saying to myself ... It was something to hold onto, more tangibly than like a physical thing, I think."

Asking simple, factual questions: Interveners went on to ask simple questions in the usual manner of 'getting to know you', enquiring where the person lived, whether they worked or were studying, and so on. This served multiple purposes, beyond establishing the facts. It gave the suicidal person something to focus on, drawing their attention away from imminent death and helping them to reconnect with themselves and their normal world. It also served gradually to build rapport between suicidal person and intervener. One participant highlighted the importance of persisting, if at first the suicidal person is unable to answer:

Survivor (1.01): "It did take quite a lot of asking ... She kept repeating the same questions ... When she asked them the first time I wasn't in a place where I could answer, but as I sort of came back into reality a bit, rather than the world inside my head ... You start to feel a bit less disconnected ... almost like it's foggy and then the fog lifts a bit, enough for you to see that there is something beyond the fog, like if the fog is suicide."

Interveners also enquired about family members and friends, and if there was someone who could be contacted (see below, *Summoning help)*.

Sharing lived experience and personal stories: As in any normal conversation, interveners reciprocated by sharing information about themselves. When interveners had their own lived experience of mental health problems, participants in both groups believed it was

For peer review only - http://bmjopen.bmj.com/site/about/guidelines.xhtml

useful to share this in order to establish credibility and trust and to provide hope of recovery. At the same time, interveners were careful to avoid focusing attention on themselves or presuming to know what the suicidal person was going through.

"Keeping it light", and not asking why: Participants in both groups expressed a strong belief that this was not the time or place to probe the reasons for wanting to die. Those in the survivor group who had been asked what had brought them to the point of suicide reported that they had been unable to talk about it, whilst others said they were glad they had not been asked. As they emerged from the 'bubble', their predominant feelings were those of ambivalence about being prevented from carrying out their plan, embarrassment and exhaustion, and their immediate need was simply for the comforting presence of another human being, not an intellectually demanding 'talking therapy' session that kept them focused on the distress:

> *Survivor (1.06): "She started saying, What's happened? Why do you feel like this? And I didn't say anything. I couldn't give her an answer. It was too complicated."*

> *Survivor (1.11): "She didn't ask why… I don't think I would have been able to answer her anyway. I would have probably bolted."*

Some interveners did ask what had brought the person to this point and some, but not all, received a response. Others reported that they had shied away from "getting in too deep" or sensed that it was not appropriate to do so, believing that exploring the precipitating events or stressors could wait until later and that making the person dwell on their problems at this point would not be useful:

For peer review only - http://bmjopen.bmj.com/site/about/guidelines.xhtml

Intervener (2.13): "I realised I didn't need to go into the why, didn't need to establish any of that stuff."

Nor was it considered helpful for a stranger to present a suicidal person with reasons for living. This was likely to be regarded as presumptuous and to provoke anger, especially when the intervener lacked lived experience of depression or suicidality.

Instead of dwelling on distress, interveners tried to keep the conversation "light" and focused on normal, everyday things, a number of them admitting that they were simply trying to buy time or alleviate their own discomfort. Survivors affirmed that this was nonetheless an effective way of "holding" them:

Intervener (2.13): "It was quite a random conversation ... I don't like silence so I just filled it with things that popped into my head."

Survivor (1.05): "He just started rambling on about his own life, which was kind of lovely. Like he was just keeping me listening even if I wasn't talking ... I could still relate to him in a way. He was saying he'd just come back from a party or something..."

Silences, and just being present: Other interveners recognised that it was possible to connect with a person without words and that silence could provide the suicidal person with a welcome respite from the intrusive and negative babble that was going on inside their own head:

For peer review only - http://bmjopen.bmj.com/site/about/guidelines.xhtml

Survivor (1.03): "She didn't really talk too much. She was just there ... And she stayed with me and bought me another cup of coffee ... and eventually the voices were stopping and I felt like I was in the moment."

The mere presence of another person was also a powerful deterrent. Survivors consistently reported that the arrival of another person made it impossible to go through with their planned suicidal act. Even at the point of crisis, they still seemed able to think of others:

Survivor (1.08): "I wanted them to just go away, but they didn't ... They were there and I didn't want to do anything in front of their little baby ... because he was old enough to know what had happened."

Embodied communication or body language was as powerful as words in establishing a non-threatening presence and conveying warmth and empathy, and included the following elements.

Proximity and positioning of self: Interveners described how they positioned themselves vis-à-vis the suicidal person, placing themselves on an equal plane (e.g. sitting or squatting) and in the same orientation (alongside and facing the same way) so as to appear friendly rather than confrontational. Maintaining a respectful distance was seen as important by those who were suicidal:

Survivor (1.09): "I remember saying, Don't come so close. I was really kind of adamant about that because this was my thing and my space."

For peer review only - http://bmjopen.bmj.com/site/about/guidelines.xhtml

Caring gestures and touch: Interveners attended to the suicidal person's physical needs, offering them a coat, a cigarette or a warm drink. Participants in both groups recognised that the warm touch of another human being could be very powerful in breaking the isolation of the suicidal person, but several survivors reported that histories of abuse had left them with a horror of physical contact and stressed the importance of asking permission before touching:

> *Survivor (1.03): "She said, Do you mind if I touch your hand? She asked me, and I can remember feeling the touch of a human person so clearly."*

> *Survivor (1.06): "Her hand was on my arm and it was warm … I was this frozen ice person, who had given up on everything, and she'd come over with sort of warm words and this physically warm touch that I didn't necessarily want to pull away from."*

General demeanour: Survivors consistently reported that the manner in which the intervener spoke to them was as important as the words spoken, if not more so. Calmness (not showing alarm), authenticity (just being themselves, being "real"), steadfastness ("not budging") and sincerity were traits that were mentioned again and again and that had the effect of making the person feel safe, valued and connected. The mere act of stopping and saying something gave a powerful signal to the person, at their lowest point, that they mattered:

> *Survivor (1.07): "The fact that you do stop makes all the difference, rather than walking past … The fact that [on three separate occasions] people stayed and talked to me … It*

For peer review only - http://bmjopen.bmj.com/site/about/guidelines.xhtml

For peer review only

was like, right now you matter and your life matters and we care what happens to you. And that was a genuine thing."

ii) Moving to a safer location

Nearly all interveners had to work out how to move the person away from the means of suicide to a safer location. Efforts to do so took one of the following three forms.

Forcible restraint: Some interventions began with a physical act of 'grabbing', designed to effect the swift and forcible removal of a person from a situation of extreme danger, before any attempt was made to engage in verbal rescue work. Whilst no-one regarded this as ideal, some survivors recognised that it had been the right thing to do in the circumstances:

Survivor (1.01): "I remember hearing two people coming up the steps … and one of them just pulled me backwards off the ledge … Had I not been pulled down, I doubt I would have engaged at all because I was so inside of myself."

The risks involved in grabbing were also clearly illustrated. In one case, two women walking on a riverside path saw a person ahead of them on the wrong side of a bridge, leaning out over the water and in extreme peril. Having reached the person in time and grabbed both her arms, they then found themselves trapped, lacking the strength to pull her back over the railing, struggling to keep hold, and able neither to engage her in conversation nor to attract the attention of passing motorists. The situation was eventually resolved, but not without injury to one of the interveners.

For peer review only - http://bmjopen.bmj.com/site/about/guidelines.xhtml

Grabbing was also seen as a purely instrumental action, which, whilst it may have been effective in saving the life on that occasion, did nothing to contribute to longer-term recovery. Interventions at the other end of the control/agency spectrum (Figure 2), which encouraged self-rescue, were deemed more likely to have an ongoing positive and protective effect.

Direct appeal ("Please come away from the edge"): This was an option when there was less urgency and it was believed that the person had the capacity to respond. Some approached it in an oblique manner:

> *Intervener (2.21):"I didn't want to confront her and say, Don't jump! … I said, Do you realise the cliff edge is really crumbly there? I'm quite worried that it might collapse. Why don't you step back a bit?"*

Indirect ("Let's go somewhere for a coffee"): Offering to buy the person a warm drink and something to eat performed multiple purposes. It could be both a simple act of kindness and validation, and also a ploy to move the person to a safer location. Either way, it was a highly effective action, serving to ease the person back into the normal, everyday world (Figure 1):

> *Intervener (2.12): "I was trying to work out how I could get her out of the tube [underground rail network] and somewhere safer … So I said, Let's go and get a cup of tea somewhere and have some cookies … And she helped herself to a cookie and there were signs she was coming to a little bit … and she sort of opened up to me then."*

For peer review only

For peer review only - http://bmjopen.bmj.com/site/about/guidelines.xhtml

iii) Summoning help

There were three potential sources of support on which interveners could call: a family member or friend nominated by the suicidal person; other passers-by, and the emergency services.

Family member or friend: Interveners typically asked about family and friends as part of “bursting the bubble,” but it was also a way to identify someone who might come and take the person home. The following example shows how a brief verbal exchange performed both tasks at once. The intervener appears initially to be trying to establish if there is someone who can be called to provide support. The suicidal person can think of no-one, but as the exchange proceeds she gradually remembers her extended family and the closeness of her social bonds (Figure 1). The sight of the photograph on her phone represented a turning point, after which she could no longer go through with the act of suicide and agreed to contact one of her daughters:

> *Survivor (1.03): “She said, Is there somebody you can ring? And I said, No, there’s nobody. And she said, Have you got any children? And I said, Yes, I’ve got two daughters. Oh, have you got a picture, she said. And I got out my phone and showed her a picture of my six grandchildren…”*

Other passers-by: Efforts to enlist help from other passers-by were rarely successful. Participants in both groups gave accounts of busy locations and people passing by, either not noticing or pretending not to notice what was happening. Several interveners made repeated and desperate attempts to signal to other passers-by but were ignored, although in some cases it transpired that a member of the passing crowd had taken it upon

For peer review only - http://bmjopen.bmj.com/site/about/guidelines.xhtml

themselves to call emergency services. However, other interveners rejected offers of help, fearing that the presence of another person would threaten the one-to-one connection and trust they were establishing with the suicidal person.

Emergency services: Interventions typically ended with the arrival of emergency services. Rail staff and highways officers had usually alerted emergency services prior to approaching the suicidal person, so that their task was simply to keep the person safe until specialist help arrived. For members of the public it was much more challenging. None of them had thought to call emergency services prior to intervening, and once they had approached and embarked on rescue work it became very difficult to do so. They feared that any move to do so, in particular any mention of police involvement, would trigger panic and flight. In some cases, the intervener asked permission to call for help and it was refused, leaving them alone and floundering. Others resorted to subterfuge or were fortunate that a member of the crowd called for help on their behalf, often unbeknown to them. Several interveners reported being unsure which service to ask for. The following example illustrates the multi-tasking nature of intervention work, the fragility of the connection with the suicidal person, and the impossibility of maintaining it whilst doing other things:

> *Intervener (2.12): "I was trying to get my phone out of my handbag without losing eye contact ... And I glanced down to unlock it and she ran ... In that split second I lost her ... So I was going after her and I was calling 999 as well, and I remember on the phone feeling quite confused because I really wasn't sure what service to be asking for ... I said, There's a lady running into the road, and they said, Well, what service do you need? And I said, I don't know..."*

For peer review only - http://bmjopen.bmj.com/site/about/guidelines.xhtml

Interveners who had worked hard to build an emotional connection with the person expressed misgivings about medicalising the situation by calling an ambulance or criminalising it by involving the police.

Endings and aftermath

Ending an intervention without a handover to emergency services or to a family member or friend was fraught with difficulty. Interveners described being desperate to extricate themselves, sometimes after several hours, but not knowing whether it was safe to let the person to go on their way and sometimes debating whether they should invite the person home with them.

Endings involving a handover were not necessarily any easier. Interveners experienced intense relief as they themselves were 'rescued', but were often left with feelings of exclusion, loss and fear of consequences for the person (especially when they were taken away in handcuffs or sectioned under the Mental Health Act). Some were left feeling disturbed by what had happened. Those who had been on their way to work at the time described the surrealness of trying to carry on *as normal* after the extraordinariness and intensity of the encounter.

For peer review only

DISCUSSION

This is the first empirical study to examine the role of passing strangers in preventing suicides. Intervention was seen to involve three main tasks: "bursting the bubble" (reconnecting with self, others and everyday world); moving to a safer location, and summoning help. Interveners accomplished these tasks in a range of ways, using both verbal and non-verbal communication and different degrees of restraint.

Those who had been suicidal (the survivor group) typically described themselves as being dissociated or "in a bubble," cut off from themselves and everything around them. Dissociative states have long been linked with suicide.[19] Orbach postulates that detachment from the body, indifference to physical pain and lack of affect are necessary in order for an individual to go through with a suicidal act. Absence of emotion is a subtle and counterintuitive clue. In this regard, suicide is like drowning insofar as the person does not look like we expect them to. A drowning person is not thrashing about and shouting for help, as commonly portrayed in film; they appear silent and still and are easily overlooked. Likewise the suicidal person rarely conforms to the stereotypical image of the anguished soul, as depicted in Munch's painting of The Scream. This is an important message to convey to the public. In an online survey of rail staff, Mackenzie and colleagues found that a high proportion of staff expected suicidal individuals to exhibit visible signs of distress,[20] suggesting that this may be a common misperception.

With few outward clues except location, interveners relied on their own 'sixth sense' that something was wrong. Some quality of oddness or incongruity triggered an alarm inside them, which they were able to hear. Thus recognition relies as much on an *internal* state

For peer review only

For peer review only - http://bmjopen.bmj.com/site/about/guidelines.xhtml

of openness, curiosity, attunement to others and attention to one's own inner voice as on any *external* signs.

Openness needs to be combined with readiness for action. The interveners in our study were ordinary people; they did not possess any special knowledge or skills. What was unusual about them was their lack of hesitancy and their disregard of the social risks involved in approaching a stranger in a public place. Most people are kept from doing so by fear of a rude rebuff and resulting embarrassment.[21] The interveners we interviewed were not fearless but, on the principle of "Feel the fear and do it anyway", they did it anyway. They also showed themselves to be resourceful, able to dig around in their personal "experience bag" and find things that worked, without necessarily knowing how or why.

Our findings suggest that people do not need to be provided with an intervention script, nor should they be afraid of saying "the wrong thing." The words the intervener spoke were not nearly as important as how they made the person feel. Survivors described interveners as having broken through the dissociative state, dispelling feelings of fear, isolation and worthlessness and making them feel safe, validated and re-connected. The basic mechanisms are depicted in Figure 3.

[INSERT FIGURE 3]

This model of effective intervention, with feeling rather than reasoning at its core, runs counter to conventional wisdom, as contained in programmes such as Applied Suicide Intervention Skills Training (ASIST)[22] and in academic literature.[8] The ASIST model

For peer review only - http://bmjopen.bmj.com/site/about/guidelines.xhtml

stresses the importance of listening closely to the person's reasons for dying and working with them to uncover reasons for living. Its focus is on verbal reasoning. Likewise, Omer and Elitzur's proposed script for a last-minute crisis intervention insists on challenging suicidal thinking. The authors compare the situation to a court of law in which the defence lawyer (the intervener) picks holes in the prosecutor's (the suicidal person's) story in an attempt to win the argument.[8] Our interveners were not attempting to win a legal battle. Nor were they trying to imitate therapists or crisis negotiators. They were just being themselves and reaching out as one human being to another. This is not to say that reasoning and verbal persuasion played no part, or that they may not be important at other stages of the suicidal process, but few of our survivors reported having been swayed by logic or argument. What they recalled most strongly about those who had helped them was their authenticity and their compassion. What made the difference was the simple fact that someone cared enough not to walk on by.

Our model may not be appropriate to other settings. A situation involving an unknown person on the point of carrying out a suicide plan in a public location is very different from other crisis settings. These include domestic or social settings, where a personal relationship exists between the two parties;[15] clinical settings, where a professional relationship and statutory responsibility for risk management exist,[23-25] and telephone counselling services, where the suicidal person has invited a talking intervention by calling a crisis line.[26 27] In these situations, the person may not yet be at the stage having formulated a suicide plan. Our study suggests that rather than there being a 'one size fits all' model of human intervention, as the ASIST training supposes, different social contexts and stages of the suicidal process may require different approaches.

For peer review only - http://bmjopen.bmj.com/site/about/guidelines.xhtml

Our findings show clearly that anyone who wants to intervene can safely do so. Last-minute intervention requires no specific learning and can be highly effective when spontaneous and unscripted. In 2018, Network Rail in partnership with Samaritans launched a campaign entitled "Small Talk Saves Lives", encouraging rail travellers simply to say 'Hello' and strike up a normal conversation if they are concerned about someone.[28] Our data confirm that this campaign message is entirely appropriate as far as the initial approach to a vulnerable person is concerned. However, it fails to recognise the intense, prolonged and taxing nature of intervention, the complex juggling acts that interveners may have to perform in the course of trying to keep someone safe, and the troubling emotions they may be left with. It is no small thing to save a life. The conundrum for public education is how to prepare people adequately for the challenges they may face without deterring them from intervening.

A set of simple public education messages is needed to encourage people to recognise and reach out to those who may be at risk of suicide in public locations. We are working with a wide range of non-academic partners to develop and disseminate these.

FIGURE LEGENDS

Figure 1: Main intervention tasks, components and interconnections

Figure 2: Control/agency spectrum

Figure 3: Proposed mechanisms of action in an effective intervention by a stranger in a public location

For peer review only - http://bmjopen.bmj.com/site/about/guidelines.xhtml

ACKNOWLEDGEMENTS

We thank the participants in both groups for sharing their stories. We are also grateful to Network Rail and Highways England for help with recruiting interveners, to Jonny Benjamin MBE, Neil Laybourn (the original *'Stranger on the Bridge'*), and the other members of our expert advisory group.

AUTHOR CONTRIBUTIONS

CO had the idea for the study and conducted public involvement. She designed the study with CA, managed the project, led the analysis and interpretation of data and wrote the manuscript. JD collected data and analysed the transcripts. Both JD and CA commented on emerging drafts of the manuscript. All authors have read and approved the final version. CO is the guarantor.

FUNDING STATEMENT

This work was supported by the Medical Research Council grant number MR/P01707X/1.

COMPETING INTERESTS

The authors declare that they have no competing interests.

ETHICS APPROVAL

Ethical approval was granted by the University of Exeter Medical School Research Ethics Committee.

For peer review only

DATA AVAILABILITY STATEMENT

We do not have consent from participants to make their transcripts publicly available. Reasonable requests for anonymised primary data for the purposes of checking the accuracy of our findings and interpretation may be sent to the corresponding author: c.v.owens@exeter.ac.uk

REFERENCES

1. Owens C, Lloyd-Tomlins S, Emmens T, et al. Suicides in public places: findings from one English county. *European Journal of Public Health* 2009;19(6):580-2.
2. Channel 4. http://www.channel4.com/programmes/the-stranger-on-the-bridge 2015 [Accessed 07.06.2019]
3. Department of Health. Preventing suicide in England: A cross-government outcomes strategy to save lives. London: HM Government 2012
4. Owens C, Hardwick R, Charles N, et al. Preventing suicides in public places: A practice resource London: Public Health England 2015. https://www.gov.uk/government/publications/suicide-prevention-suicides-in-public-places.
5. Cox G, Owens C, Robinson J, et al. Interventions to reduce suicides at suicide hotspots: A systematic review. *BMC Public Health* 2013;9(13):214.
6. Pirkis J, Spittal M, Cox G, et al. The effectiveness of structural interventions at suicide hotspots: a meta-analysis. *Int J Epidemiol* 2013;42(2):541-8.
7. Pirkis J, Too L, Spittal M, et al. Interventions to reduce suicides at suicide hotspots: a systematic review and meta-analysis. *The Lancet Psychiatry* 2015;2(11):994-1001.
8. Omer H, Elitzur A. What would you say to the person on the roof? A suicide prevention text. *Suicide Life Threat Behav* 2001;31(2):129-39.

For peer review only - http://bmjopen.bmj.com/site/about/guidelines.xhtml

9. World Health Organization. Preventing Suicide: A Resource for Police, Firefighters and Other First Line Responders. Geneva: World Health Organization 2009. http://www.who.int/mental_health/prevention/suicide/resource_firstresponders.pdf [Accessed 07.06.2019]

10. Lanceley F. On-Scene Guide for Crisis Negotiators (2nd edition). Boca Raton, Fl: CRC Press 2003.

11. Greenstone J. Teen Suicide: Negotiating with the Young Suicidal Actor. *Journal of Police Crisis Negotiations* 2008;3(2):61-64. doi: 10.1300/J173v03n02_07

12. Fowler W, Maguire J. Dealing with Defenestrators: Immediate Interventions. *Journal of Police Crisis Negotiations* 2001;1(2):41-51.

13. Holloway W, Jefferson T. Doing Qualitative Research Differently: Free Association, Narrative and the Interview Method. London: Sage 2000.

14. Wengraf T. Qualitative Research Interviewing: Biographic Narrative and Semi-Structured Methods. London: Sage 2001.

15. Owens C, Owen G, Belam J, et al. Recognising and responding to a suicidal crisis in the family and social network: qualitative study *BMJ* 2011;343:d5801.

16. Cannon S. Social research in stressful settings: difficulties for the sociologist studying the treatment of breast cancer. *Sociol Health Illn* 1989;11(1):62-77.

17. Lee RM. Doing research on sensitive topics. London: Sage 1993.

18. Holstein K, Gubrium J. Active interviewing. In: Silverman D, ed. Qualitative Research: Theory, Method and Practice. London: Sage 1997.

19. Orbach I. Dissociation, physical pain and suicide: A hypothesis. *Suicide and Life-Threatening Behavior* 1994;24(1):68-79.

For peer review only - http://bmjopen.bmj.com/site/about/guidelines.xhtml

20. Mackenzie J, Borrill J, Hawkins E, et al. Behaviours preceding suicides at railway and underground locations: a multimethodological qualitative approach. *BMJ Open* 2018;8:e021076. doi: doi: 10.1136/bmjopen-2017-021076

21. Goffman E. Behavior in Public Places: Notes on the Social Organization of Gatherings. New York: Free Press 1963.

22. LivingWorks Education. https://www.livingworks.net/programs/asist/ 2014-16 [Accessed 07.06.2019] [

23. Appleby L, Morriss R, Gask L, et al. An educational intervention for front-line health professionals in the assessment and management of suicidal patients (The STORM Project). *Psychological Medicine* 2000;30(4):805-12.

24. Hendin H, Maltsberger J, Lipschitz A, et al. Recognizing and responding to a suicide crisis. *Suicide Life Threat Behav* 2001;31(2):115-28.

25. Department of Health. Best Practice in Managing Risk: Principles and Evidence for Best Practice in the Assessment and Management of Risk to Self and Others in Mental Health Services. London: Department of Health 2009. https://assets.publishing.service.gov.uk/government/uploads/system/uploads/attachment_data/file/478595/best-practice-managing-risk-cover-webtagged.pdf [Accessed 07.06.2019].

26. Mishara B, Chagnon F, Daigle M, et al. Which helper behaviors and intervention styles are related to better short-term outcomes in telephone crisis intervention? Results from a Silent Monitoring Study of Calls to the U.S. 1-800-SUICIDE Network. *Suicide Life Threat Behav* 2007;37(3):308-21.

27. Gould M, Cross W, Pisani A, et al. Impact of Applied Suicide Intervention Skills Training on the National Suicide Prevention Lifeline. *Suicide and Life Threatening Behavior* 2013;43(6):676-91. doi: 10.1111/sltb.12049

For peer review only - http://bmjopen.bmj.com/site/about/guidelines.xhtml

28. Samaritans. https://www.samaritans.org/media-centre/our-campaigns/small-talk-saves-lives 2018. [Accessed 07.06.2019]

For peer review only

Figure 1: Main intervention tasks, components and interconnections

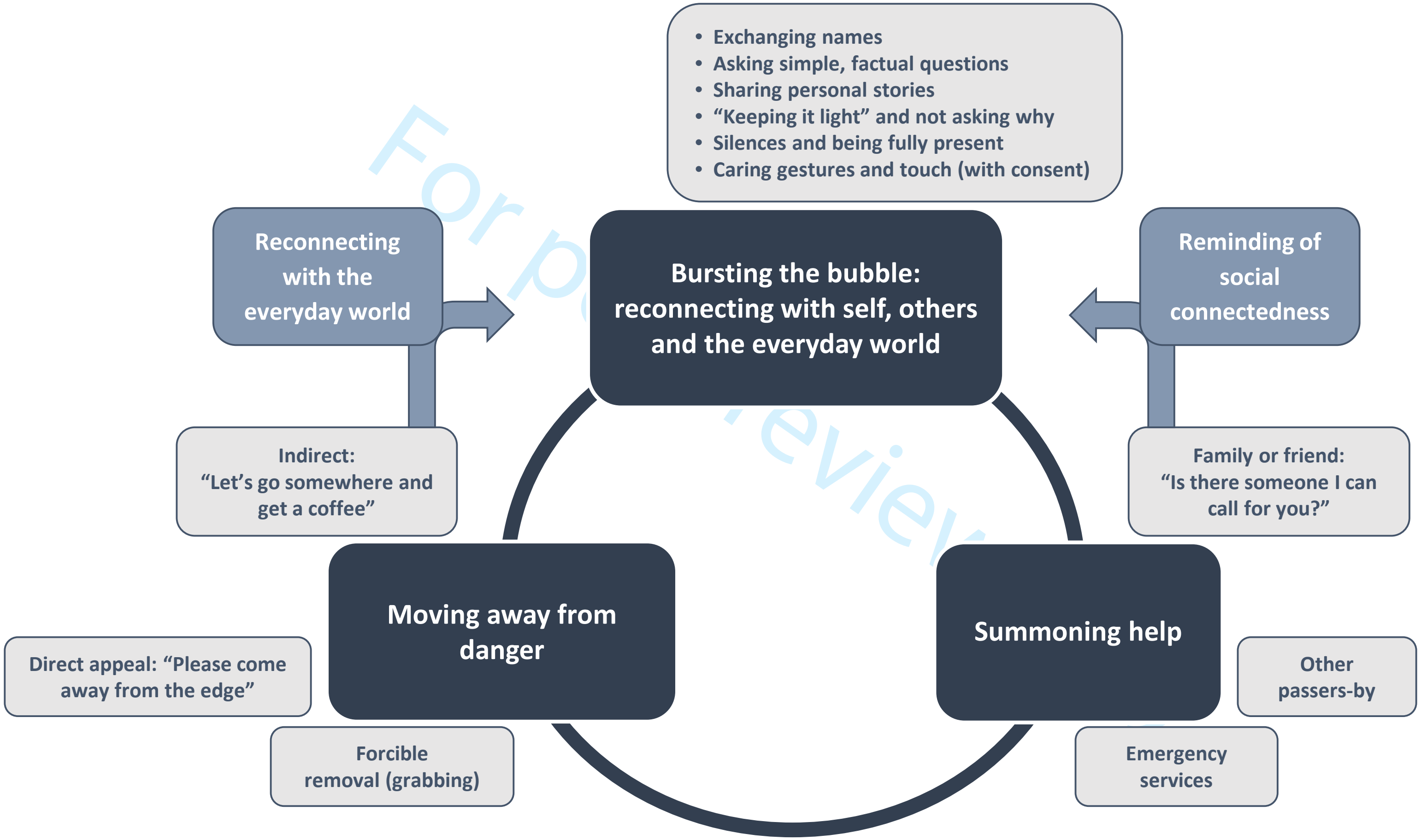

Figure 2: Control/agency spectrum

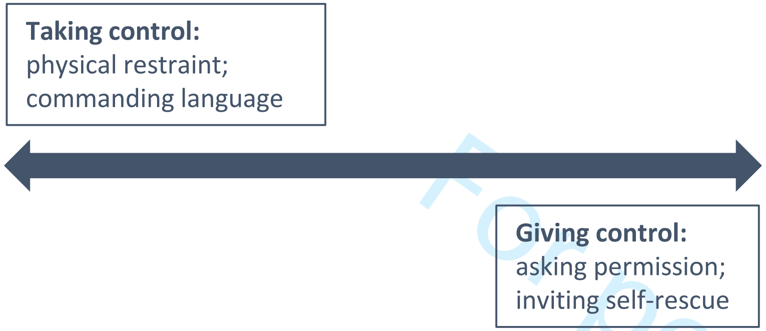

Figure 3: Proposed mechanisms of action in an effective intervention by a stranger in a public location

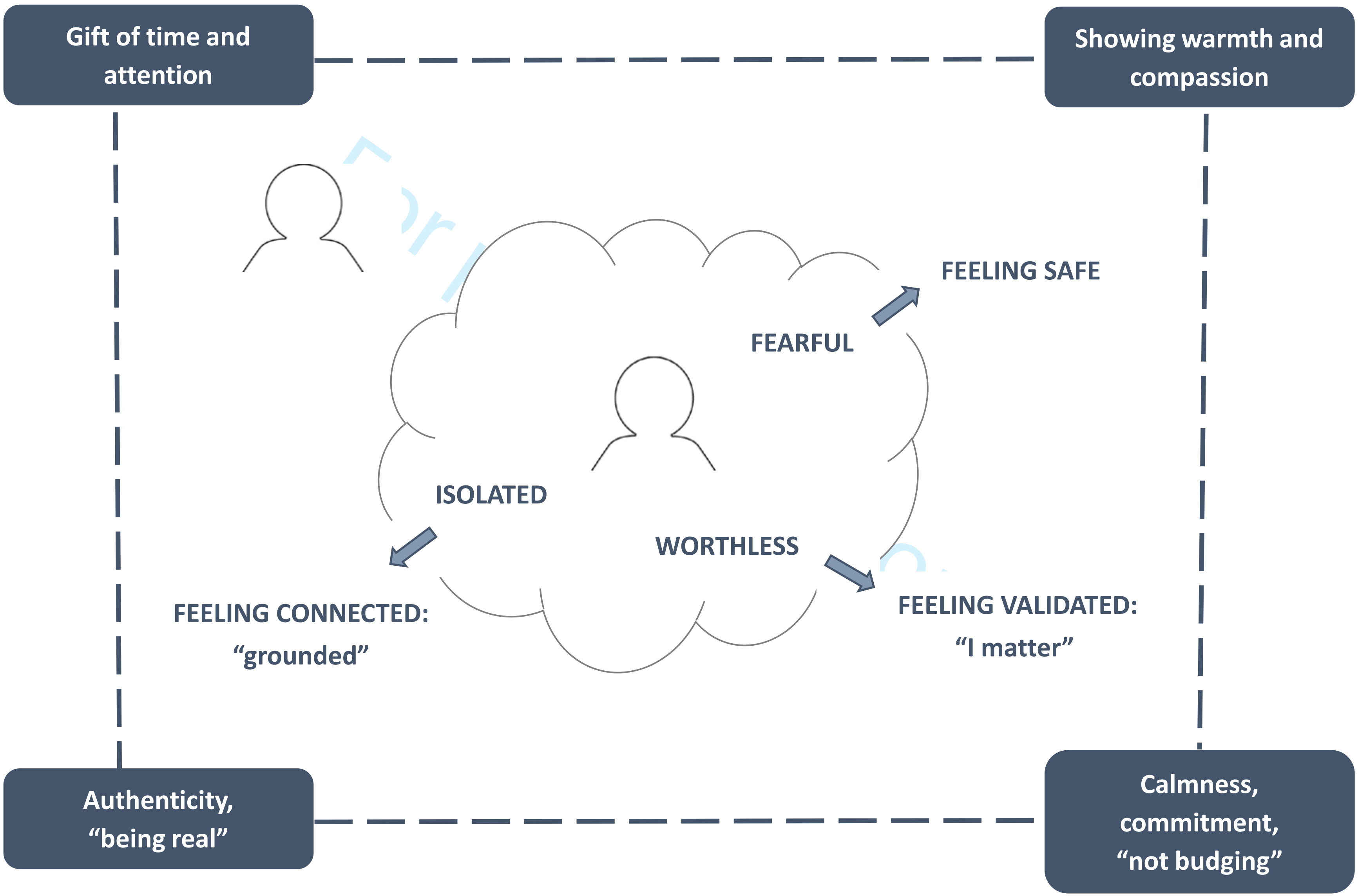

Topic guide
Group 1: Those with experience of being stopped from taking their own lives

Checklist:
- Information sheet and consent form
- Tape recorder
- Spare batteries
- Biscuits or cake
- Turn phone to silent

Preamble:
- *Thanks for coming forward; importance of understanding the situation from your point of view*
- *Brief recap of aims of study*
- *Recap of confidentiality*
- ***Ask directly** about current mental state and suicidality: "Have you had any thoughts of suicide recently [in the last week or two]? How are you feeling right now?" If currently receiving psychiatric care, do they have a letter from psychiatrist? If in any doubt, advise not to proceed.*
- *Stopping rule: "I realise that talking about that time will be difficult for you. You are completely free to stop anytime you wish, or you can take a break and then decide whether or not to carry on. Please tell me if talking about it triggers thoughts of harming yourself or makes you feel in any way unsafe. If that happens, you **must** stop."*
- *Sign consent form*
- *Brief outline of interview*

Opening free narrative:

"So I know that you came close to taking your own life in [month/year]. In your own time, please tell me what led up to it and what happened on that particular day."

Prompts and suggested follow-up. Ensure the following are fully explored:

Setting:
- Clarify where exactly it took place
- Why there (choice of location)? Why that method of suicide? Had you thought about other places and possibly other methods of ending your life? How carefully planned was it?
- How did you get there? How long did the journey take?
- What was going through your mind when you got there? [Probe: thinking and feeling]

Intent:
- How much did you want to die?
  or
  Did you think someone might try to stop you? Was any part of you hoping that might happen?

Intervention:
- Who approached you and how?
- What was the first thing they said to you? Then what…?
- How did they say it? [Probe: body language, positioning, eye contact, touch, other non-verbal signals, pace and timing, general manner and tone of voice.
- How did it make you feel?

For peer review only

- What other actions did they take, including calling emergency services?
- What helped most, made the most difference?
- What wasn't helpful, irritated or angered you?
- What sort of things should people avoid saying or doing?

- What message would you like to give to anyone who is unsure about whether or not to intervene?

- Thinking more broadly about prevention and about that particular place, apart from someone stepping in and talking to you, is there anything else that might have prevented you from going through with the suicide? [<u>Prompt if necessary</u>: e.g. higher parapet on bridge, fencing on railways, emergency telephone…]

<u>Participant's background</u>

- Age
- Ethnicity
- Region (London; South East; South West etc)
- Employment status [employed; unemployed; student; homemaker; carer; retired…]
- If working, what do they do?

- Have you kept in touch with the person who helped you? If so, how would you feel about us possibly contacting them and asking them if they would be interviewed?

- Is there anything else you'd like to add?

<u>Close:</u>

- *Thanks again etc.*
- *Offer Amazon voucher and check email address*
- *Tell them they can get in touch if they think of anything afterwards that they'd like to add or change*
- *Ask if they'd like to receive further information about the study and a summary of the findings*
- *Ask if they know others who've been in similar situation who might want to take part*
- *Ask if they have any ideas about how to advertise study.*

<u>Debrief/safety check:</u>

- *How are they feeling right now? **<u>Ask directly</u>**: "Has talking about that day made you feel like harming yourself again?"*
- *Do they have plans for the rest of the day? Where are they planning to go [immediately after the interview]; are they meeting up with anyone? Offer to stay with them until then, or ask them to text you when they get there*
- *Give details of organisations to contact for support if they become upset later*
- *<u>If they give any cause for concern</u>, ask if you can accompany them to a place of safety (a trusted friend's house or A&E).*

For peer review only

Topic guide
Group 2: Those with experience of intervening

Checklist:
- Consent form
- Tape recorder
- Spare batteries
- Biscuits or cake
- Turn phone to silent

Preamble:
- *Thanks for coming forward; importance of understanding the situation from your point of view*
- *Brief recap of aims of study*
- *Recap of confidentiality*
- *Possible that it may be upsetting to recall what happened. If that happens, please tell me and we will stop the interview. You are completely free stop anytime you wish, or you can take a break and then decide whether or not to carry on.*
- *Sign consent form*
- *Brief outline of interview*

Opening free narrative:

"So I know that in [month/year] you saw someone in a public place who you thought was at risk of harming themselves and that you tried to help them. In your own time, please tell me what happened on that particular day."

Prompts and suggested follow-up. Ensure the following are fully explored:

Setting
- Clarify where exactly it took place
- What exactly were you doing when you first noticed the person? Where were you going? Paint a picture of the scene.

Recognition
- What was the first thing you noticed about them?
- How quickly did you realise that they might be in need of help of some sort?
- Did you immediately recognise that they were at risk of harming themselves?
- What was it that enabled you to know that? [Probe: features, appearance, behaviour of suicidal person **plus** participant's own background /previous experience or exposure]

Preparedness
- What went through your mind when you realised that?
- How quickly did you decide to approach them, and how did you make that decision?
- Did you know what to say or do, or were you just 'winging it'?
- How confident did you feel that you could help them? [Probe for: fears about intervening; feelings about competence]

For peer review only

Intervention
- What was the first thing you said or did?
- Then what…?
- What was going through your mind while it was all happening? How were you feeling?
- Do you think you made any mistakes? Anything you regret saying or doing? If so, what?
- What would you do differently another time?
- Do you have any thoughts about what was most helpful?

Personal consequences:
- What impact has it had on you? [Probe: immediate; longer-term, depending on how long ago; future]
- Would you do it again? Why/why not?
- What message would you give to anyone who is unsure about whether or not to intervene in a situation like the one you encountered?

Training
- If not already mentioned: Have you received any mental health or suicide prevention training? If so, what and when?
- Do you think members of the public should receive basic suicide prevention training, in the same way that they get first aid training? What form do you think it should take?
- What sort of information would have been helpful to you in the situation you found yourself in?

Participant's background
- Age
- Ethnicity
- Region (London; South East; South West etc)
- Employment status [employed; unemployed; student; homemaker; carer; retired…]
- If working, what do they do?

- Have you kept in touch with the person you helped? If so, how would you feel about us possibly contacting them and asking them if they would be interviewed?

- Is there anything else you'd like to add?

Close:
- *Thanks again etc.*
- *Offer Amazon voucher and check email address*
- *Tell them they can get in touch if they think of anything afterwards that they'd like to add or change*
- *Ask if they'd like to receive further information about the study and a summary of the findings*
- *Ask if they have any links with others in similar situation*
- *Ask if they have any ideas about how to advertise the study.*

Safety check:
- *How are they feeling right now? Has it been upsetting recalling the events of that day?*
- *If so, check plans for the rest of the day? Do they have someone they can meet up with?*
- *Give details of organisations to contact for support*

For peer review only

Consolidated criteria for reporting qualitative studies (COREQ): 32-item checklist

**Guide questions/description**	**Authors' response**
Domain 1: Research team and reflexivity	
Personal Characteristics<br>1. Interviewer/facilitator. Which author/s conducted the interview ~~or focus group~~?	Reported in manuscript, p.8 (all page numbers refer to revised manuscript)
2. Credentials. What were the researcher's credentials? E.g. PhD, MD	Master's degree and PhD
3. Occupation. What was their occupation at the time of the study?	University Research Fellow
4. Gender. Was the researcher male or female?	Female
5. Experience and training. What experience or training did the researcher have?	Extensive track record in use of qualitative research methods, including PhD and 10 years' post-doctoral experience.
Relationship with participants<br>6. Relationship established. Was a relationship established prior to study commencement?	Email and telephone contact prior to interview
7. Participant knowledge of the interviewer. What did the participants know about the researcher? e.g. personal goals, reasons for doing the research	The interviewer shared relevant personal information during preliminary conversations.
8. Interviewer characteristics. What characteristics were reported about the interviewer/facilitator? e.g. Bias, assumptions, reasons and interests in the research topic	Insufficient space to report
Domain 2: study design	
Theoretical framework<br>9. Methodological orientation and Theory. What methodological orientation was stated to underpin the study? e.g. grounded theory, discourse analysis, ethnography, phenomenology, content analysis	Inductive thematic analysis, described in detail on p.8-9
Participant selection<br>10. Sampling. How were participants selected? e.g. purposive, convenience, consecutive, snowball	There are a limited number of people in the general population who meet the inclusion criteria and they are impossible to identify except by advertising the study and hoping they will come forward. This was the only possible sampling strategy.
11. Method of approach. How were participants approached? e.g. face-to-face, telephone, mail, email	Potential participants expressed interest by emailing a project mailbox. They were then contacted by return email, with a participant information leaflet attached, and were invited to receive a telephone call to discuss the study in more detail and ask questions. Once the researcher was confident that they were eligible to participate and happy to proceed, interview arrangements were made. Written

For peer review only

	consent was taken on the day of interview.
12. Sample size. How many participants were in the study?	Reported in manuscript, p.10
13. Non-participation. How many people refused to participate or dropped out? Reasons?	n/a
Setting<br>14. Setting of data collection. Where was the data collected? e.g. home, clinic, workplace	Reported in manuscript, p.8
15. Presence of non-participants. Was anyone else present besides the participants and researchers?	No
16. Description of sample. What are the important characteristics of the sample? e.g. demographic data, date	Reported in manuscript, p.10 and Table 1
Data collection<br>17. Interview guide. Were questions, prompts, guides provided by the authors? Was it pilot tested?	Reported in manuscript, p.8. Interview guides uploaded as supplementary files.
18. Repeat interviews. Were repeat interviews carried out? If yes, how many?	No repeat interviews
19. Audio/visual recording. Did the research use audio or visual recording to collect the data?	Reported in manuscript, p.8
20. Field notes. Were field notes made during and/or after the interview ~~or focus group~~?	Yes
21. Duration. What was the duration of the interviews ~~or focus group~~?	Reported in manuscript, p.8
22. Data saturation. Was data saturation discussed?	Reported in manuscript, p.7
23. Transcripts returned. Were transcripts returned to participants for comment and/or correction?	No. It was not considered ethical for the 'survivor' group to read accounts of their own past suicidal crises without a researcher present to offer support should they become distressed or find the accounts triggering.
Domain 3: analysis and findings	
Data analysis<br>24. Number of data coders. How many data coders coded the data?	One researcher (JD) coded all transcripts using NVivo; another researcher (CO) coded independently using non-computerised methods and also checked JD's NVivo coding of several full transcripts.
25. Description of the coding tree. Did authors provide a description of the coding tree?	Available from the authors
26. Derivation of themes. Were themes identified in advance or derived from the data?	Reported in manuscript, p.8-9
27. Software. What software, if applicable, was used to manage the data?	Reported in manuscript, p.9
28. Participant checking. Did participants provide feedback on the findings?	Yes. We presented findings on three occasions to an expert advisory group comprising people with lived experience and a range of external stakeholders. We also sent a summary of findings to all

For peer review only

	participants and invited their comments. Reported in manuscript, p.9
Reporting<br>29. Quotations presented. Were participant quotations presented to illustrate the themes / findings? Was each quotation identified? e.g. participant number	Yes to both questions. Extensive illustrative quotations were presented. These were identified by participant group (intervener or survivor) and individual number.
30. Data and findings consistent. Was there consistency between the data presented and the findings?	Yes, we would hope so.
31. Clarity of major themes. Were major themes clearly presented in the findings?	Yes, we would hope so. Major themes are listed on p.11
32. Clarity of minor themes. Is there a description of diverse cases or discussion of minor themes?	Yes, sub-themes are described on pp.15-26. Major and minor themes are also represented in Figure 1.

For peer review only

BMJ Open

Intervening to prevent a suicide in a public place: A qualitative study of effective interventions by lay people

Journal:	*BMJ Open*
Manuscript ID	bmjopen-2019-032319.R2
Article Type:	Original research
Date Submitted by the Author:	01-Oct-2019
Complete List of Authors:	Owens, Christabel ; University of Exeter, College of Medicine and Health<br>Derges, Jane; University of Exeter, College of Medicine and Health<br>Abraham, Charles; University of Melbourne, School of Psychological Sciences
**Primary Subject Heading**:	Public health
Secondary Subject Heading:	Mental health, Qualitative research
Keywords:	Suicide & self-harm < PSYCHIATRY, PUBLIC HEALTH, QUALITATIVE RESEARCH, MENTAL HEALTH

SCHOLARONE™
Manuscripts

Intervening to prevent a suicide in a public place:

A qualitative study of effective interventions by lay people

Authors:

Christabel Owens,* University of Exeter Medical School, Exeter, UK

Jane Derges, University of Exeter Medical School, Exeter, UK

Charles Abraham, School of Psychological Sciences, University of Melbourne, Australia

*** Corresponding author:**

Professor Christabel Owens, University of Exeter Medical School, College House,

St Luke's Campus, Exeter EX1 2LU, United Kingdom.

Tel: +44 (0)1392 726006

Email: c.v.owens@exeter.ac.uk

For peer review only

ABSTRACT

Objectives: Many suicides take place in public locations, usually involving jumping from high places or use of transport networks. Previous research has largely focused on the effectiveness of physical barriers at frequently-used locations. There have been no studies of human intervention to prevent suicides in public places. The aim of this study was to identify the core components of an effective intervention by a member of the public.

Design and methods: We conducted in-depth qualitative interviews with people who have either been prevented by a stranger from taking their own life in a public location (n=12) or intervened to prevent a stranger from taking their own life in a public location (n=21). Collectively, the two groups narrated 50 incidents of suicide rescue. We analysed interview transcripts using inductive thematic analysis.

Results: Suicidal people typically displayed no visible distress, describing themselves as being dissociated or "in a bubble." Intervention was seen to involve three main tasks: "Bursting the bubble" (reconnecting with self, others and everyday world); Moving to a safer location, and Summoning help. We show how interveners accomplished these tasks in a range of ways, using both verbal and non-verbal communication and different degrees of restraint.

Conclusions:

This is the first empirical study to examine the role of passing strangers in preventing suicides in public places. It shows that no specialist skills are needed. Interveners were ordinary people, distinguished only by a high level of social awareness, combined with a

For peer review only - http://bmjopen.bmj.com/site/about/guidelines.xhtml

readiness for social action. The findings also suggest that people do not need a script and should not be afraid of saying “the wrong thing.” What interveners said was much less important than how they made the suicidal person feel, namely safe, connected and validated (“I matter”). Interveners did this simply by being themselves, responding with authenticity, calmness and compassion. Members of the public need to be encouraged to recognise and reach out to those who may be at risk of suicide in public locations, but should be prepared for a prolonged and intense encounter that may leave them with troubling emotions.

For peer review only - http://bmjopen.bmj.com/site/about/guidelines.xhtml

STRENGTHS AND LIMITATIONS OF THIS STUDY

- This is the first empirical study to examine the role of passing strangers in preventing suicides.
- We included the perspectives of both interveners and those whose lives had been saved by the intervention of a stranger and found a very high level of agreement between them as to what worked, giving confidence in our conclusions about effective interventions.
- Although we did not specify that interventions must have been successful, our intervener group did not include any individuals who had failed to avert a suicide, so we are unable to draw any conclusions about ineffective interventions.
- We allowed a wide range in the length of time between incident and interview (from less than one year to more than ten years), extending our original upper limit of five years in some cases, after careful screening to assess the quality of recall; all participants were able to give extremely detailed accounts, which we had no reason to doubt.
- Our model of effective suicide crisis intervention may be culturally-specific and may not be suited to other countries, particularly those in east Asia that have much higher incidence of suicides in public places.

For peer review only

INTRODUCTION

Suicide accounts for around 6,000 UK deaths each year, up to a third of which occur in public locations.[1] These may be more easily preventable than those that occur in the privacy of the home, because of the potential for a chance passer-by to make a last-minute intervention. There is compelling anecdotal evidence that lives can be saved by passing strangers acting on the spur of the moment. *The Stranger on the Bridge* was an award-winning documentary, broadcast on UK television in 2015, which told the story of one such intervention and captured global media attention.[2]

Preventing suicides in public places occupies a key place in England's national suicide prevention strategy.[3] In 2015 Public Health England published guidance for local authorities to help them identify sites and structures that might be used for suicides and take action to prevent deaths at those locations.[4] These typically include sites that provide opportunity for jumping from a height (bridges, high buildings and cliffs) and sections of the road and railway networks. Previous research has largely focused on the effectiveness of installing physical barriers at such locations to restrict access to means.[5-7] There are no studies of human intervention, and no evidence-based guidance for members of the public. The literature contains only one published article on the topic of what a potential helper might say in a last-minute crisis intervention.[8] Based on the authors' clinical experience rather than research evidence, it provides a suggested script that is long, verbose and contains no clear messages for the general public; nor has it been tested. The World Health Organization (WHO) has published guidance for emergency services personnel responding to suicidal crises,[9] but the advice is very general and does not differentiate between crises occurring in private and in public places. There is an extensive literature on police crisis negotiation, some of which deals with suicide

For peer review only - http://bmjopen.bmj.com/site/about/guidelines.xhtml

intervention,[10-12] but this is either too general, in the manner of the WHO document, or too specialized, describing 'tricks of the trade' that could not be safely recommended to lay people.

We were not interested in the part played by health professionals or members of the emergency services, whose jobs include suicide crisis intervention, but in ordinary citizens who happen to be passing by. The aim of this study was to identify the core components of an effective intervention by a stranger in a public place.

For peer review only - http://bmjopen.bmj.com/site/about/guidelines.xhtml

METHODS

Design

We conducted a qualitative interview study in order to understand intervention by a passing stranger from the perspective of both parties.

Participants and recruitment

Participants were eligible for inclusion if they were aged 18 or over and had experience of either of the following:

- Being stopped from taking their own life in a public location by a passing stranger (Survivors);
- Trying to stop someone they did not know from taking their own life in a public location (Interveners). This group included members of the general public and members of staff in non-health agencies whose work at public locations brings them into contact with suicidal individuals, such as railway staff.

A public location was defined as anywhere outside their own or someone else's home. For both groups, we placed advertisements on the websites of mental health and suicide prevention charities and used the social media accounts (Facebook and Twitter) of non-academic partners to invite people with relevant experience to come forward. Interveners were also sought via relevant agencies, including Network Rail, Highways England and bridge authorities. We continued recruiting until each group contained a reasonable degree of diversity in respect of age, gender and ethnicity and we were confident that no new insights were emerging (data saturation).

Interviews

For peer review only - http://bmjopen.bmj.com/site/about/guidelines.xhtml

We conducted in-depth interviews with both groups, beginning with an invitation to give an uninterrupted account: "Please tell me in your own words as much as you can remember about [the event in question]." This free narrative approach affords insight into what is uppermost in the participant's mind.[13-15] It also allows them to disclose information at their own pace, thereby increasing their sense of safety when talking about difficult or sensitive subjects.[16 17] During the narrative phase, the interviewer [JD, an experienced qualitative researcher] assumed the role of active listener. She then proceeded to ask open-ended questions, following up any points requiring clarification and probing for further detail where necessary.[18] Separate interview guides for each group were used to ensure that all topics of interest were covered and that safety protocols were followed [available as supplementary files]. Interveners reflected in detail on what they said/did and how key decisions were made. Survivors described what the intervener said/did and how it affected them, allowing us to examine mechanisms of action.

All interviews with survivors were conducted face-to-face, mostly in participants' homes, and lasted 1-2 hours. Interviews with interveners were conducted face-to-face (n=15), by telephone (n=4) or Skype (n=2). All participants gave written consent. Interviews were audio-recorded, transcribed verbatim and anonymised.

Analysis

Data collection and analysis were carried out concurrently to allow for progressive focusing of interviews and exploration of emerging ideas. Two investigators [CO and JD] independently read the first three transcripts from each group (n=6) before meeting to discuss candidate themes and analytic strategy. They then worked line-by-line through

For peer review only - http://bmjopen.bmj.com/site/about/guidelines.xhtml

six further transcripts, noting and naming specific features of the situation, including: characteristics of the actors; discrete intervention elements (verbal and non-verbal) and their effects on the suicidal person; constraints, complicating factors and tipping points. These were used to construct a list of categories, with definitions, that were used for coding the entire data set. Coding, sorting and retrieval were carried out using NVivo software. We then carefully examined all the coded material, exploring each category and its relationship to others, visually mapping intervention elements, and working via a series of iterations and data checks towards our final interpretive model.

Patient and public involvement

We designed the study in consultation with people with relevant lived experience, representatives from suicide prevention charities and the transport industry. They were instrumental in recruiting participants and subsequently commenting on findings and interpretation. We will continue to work with them as we start to frame key public education messages. A lay summary of results was sent to participants, who were also invited to help with future message development work.

For peer review only

For peer review only - http://bmjopen.bmj.com/site/about/guidelines.xhtml

RESULTS

Characteristics of the sample

We recruited 12 people who had been stopped from taking their own life in a public place (Survivors): nine female and three male, with ages ranging from 19-64. Four of them had been stopped on more than one occasion (one twice and three on three occasions), giving a total of 19 accounts of successful intervention.

The Intervener group contained 21 individuals: nine male and twelve female, also ranging in age from 19-64. This number was made up of 13 members of the general public, six railway workers[a] (two of whom were off-duty at the time) and two highways officers. Between them they had conducted 31 interventions, all successful. Most of the railway workers and one of the highways officers had intervened successfully on more than one occasion. Three members of the public had conducted more than one rescue. One participant was included in both groups because, having been stopped from taking her own life in a public place, she went on to conduct two successful interventions. The members of the public included students, teachers/lecturers, youth leaders, church and charity workers, civil servants, office workers and an actor. Several of them had lived experience of mental health problems. They recounted how, during the intervention, they drew on a miscellany of skills and scraps of knowledge that they had acquired in other spheres, including work and leisure pursuits. One used the metaphor of an 'experience

[a] The railway staff were all Mobile Operations Managers, whose role is to deal with a range of incidents on the railway network, including threatened suicides and fatalities. They differed from members of the general public insofar as: a) they were often acting on a referral, so had some time to prepare; b) five out of the six had received basic suicide intervention training from Samaritans; c) they had responsibilities beyond caring for the suicidal person, including restoring train services as quickly as possible, and d) they had organisational arrangements in place for managing such incidents, including direct links to the British Transport Police. Our rationale for including them in the intervener group was that they were responding as 'lay people', i.e. not as mental health professionals, to a threat of suicide by a stranger in a public location.

For peer review only - http://bmjopen.bmj.com/site/about/guidelines.xhtml

bag' that she carried around with her and had raided for ideas and possible strategies to use while struggling on two different occasions to keep a suicidal person safe.

Of the 50 interventions recounted (19 by survivors and 31 by interveners), 27 occurred on the rail and road networks (including bridges over road or railway). A further 18 involved other types of bridge or high places. Characteristics of participants and settings are shown in Table 1.

Table 1: Participants and settings

	Survivor group (n=12)	Intervener group (n=21)
Age range	19-64	19-64
Gender:		
Female	9	12
Male	3	9
Ethnicity:		
White British/European	10	21
Asian/Asian British	2	-
Black/Black British	-	-
Other ethnic groups	-	-
Mixed/Multiple ethnicity	-	-
Location:	(n of incidents =19)	(n of incidents =31)
Rail/underground/bridge over railway	4	14
Road network/bridge over road	2	8
River/bridge over river	7	4
High building	3	0
Cliffs	2	3
Other	1	2
Time since incident:	(n=19)	(n=31)
1 year or less	7	7
2 – 5 years	8	10
6 - 10 years	2	12
More than 10 years	2	2

We present our findings under the following headings: Recognition; Three intervention tasks ("Bursting the bubble," Moving to a safer location, and Summoning help), and Endings and aftermath.

For peer review only - http://bmjopen.bmj.com/site/about/guidelines.xhtml

Recognition

The prerequisite for intervention is recognition. Interveners recognised the person as being at risk or in need of help primarily by their location. The person either appeared to have placed themselves in immediate physical danger or was judged to be out of place, giving rise to curiosity or concern:

> *Intervener (2.06):"There was a person standing there in a hoodie, sort of facing the railway and looking over the side ... and I took a couple of steps and thought, Hang on, what's she doing there? I did literally stop and think, Hang on, this is odd."*

Survivors also acknowledged the oddness of their positioning:

> *Survivor (1.01):"I went very close to the edge of the platform and sat with my feet over the edge ... quite hunched up, possibly my hands over my ears and my eyes closed. I mean, it's not generally a position people take when they're waiting for a train."*

There were few clear behavioural clues and rarely anything in their mode of dress or appearance to mark them out. What was striking in interveners' accounts of what they saw was the absence of visible emotion. They described the suicidal person as looking "vacant", "glazed over", "zoned out" or "as if no-one was at home", but not distressed.[b] This was corroborated in the accounts of survivors, who described themselves as having

[b] There were two instances recounted by interveners in which the person was sobbing inconsolably. In both cases the person spoke of wanting to end their life, but neither was in a high-risk location or in possession of a means of suicide, suggesting that they were not about to carry out a plan. This was confirmed by a survivor (1.01), who said, *"I wouldn't even be able to walk to the station if I was crying that much. It would be when I've run out of tears ... and I just feel empty and numb and disconnected. That's when I'm much more likely to act."*

For peer review only - http://bmjopen.bmj.com/site/about/guidelines.xhtml

moved into a space beyond emotion: “a weird sort of surreal, unfeeling state” in which they were “completely numb”, “frozen” or “dissociated”, cut off from themselves, others and the everyday world, to the point where they believed they were invisible. They were not inviting rescue:

Survivor (1.11): “It didn’t occur to me that people would stop and take notice. I was very much of this mindset that I was invisible and no-one would see me.”

This state of dissociation was described as being “in a bubble”.

Interveners, on the other hand, appear to have been characterised by a state of openness to what was going on around them and an attunement to human distress even when it was not manifest. Some reflected that this had been acquired from parents with a strong sense of social justice or through experience in particular settings, such as working as a bar manager: “They teach you to spot trouble before it happens” (Intervener 2.15). This participant had been walking across a bridge with his partner when they encountered a person preparing to take her own life. He knew immediately that something was amiss, whilst his partner was unaware of anything until he started to intervene.

Three intervention tasks

We identified three main tasks involved in intervention:

i) “Bursting the bubble” (reconnecting with self, others and the everyday world);

ii) Moving to a safer location;

iii) Summoning help.

For peer review only - http://bmjopen.bmj.com/site/about/guidelines.xhtml

The three tasks are interconnected, as shown in Figure 1 and described below. Interventions were typically complex and interveners' accounts, particularly those of general public members who did not have organisational structures around them, show them to be frantically multi-tasking during the course of the encounter, trying to think ahead and plan several possible moves like chess players. Occasionally they managed to achieve several purposes through a single speech act or gesture.

[INSERT FIGURE 1]

The order in which they approached the tasks and the manner of intervention was influenced by a number of factors, including: the immediacy of danger; clarity of suicidal intent; the capacity of the suicidal person to interact verbally; the intervener's personality, and whether they were in a position of authority and responsibility. Interventions and their constituent parts fell on a spectrum according to the degree of control assumed by the intervener (Figure 2). At one end were interventions involving forcible restraint and authoritative language, often necessitated by the perceived urgency of the situation. At the opposite end, some interveners were acutely mindful of the suicidal person's right to self-determination and need to feel in control of their own destiny. Accordingly, they asked permission before taking any action and used invitations and appeals, calling on the person to rescue themselves and allowing them to keep all their options open:

Survivor (1.05): "He came up behind and he said, Look, I don't want to stop you from doing anything but I'd like it if you could come over the barrier ... Just step on over, it's OK ... He said, I'm not going to take you away from here, you can stay here if you want ...

For peer review only - http://bmjopen.bmj.com/site/about/guidelines.xhtml

It was like, he's not forcing me to do anything. I can still do it ... and that was important for me."

[INSERT FIGURE 2]

i) "Bursting the bubble"

Both interveners and survivors used the metaphor of the bubble and described intervention in terms of bringing the suicidal person out of their trance-like, dissociated state and back into the present moment, reconnecting them with themselves, others and the everyday world. This emotional labour formed the heart of most of the interventions we studied. It was predominantly verbal in nature, although embodied communications sometimes also played a key part.[c]

Initial approach: The work began with the intervener establishing their presence. Most approached cautiously and started with some kind of question, such as:

"Are you OK?"

"Can I ask what you're doing there?"

"You seem to be in a spot of trouble. Can I help?"

These apparently simple enquiries did not always elicit a straightforward answer. The intervener still needed to exercise personal judgement, and quickly, as in the following example:

[c] The term embodied communication refers to instances in which one social actor conveys a message to another by means of the body or bodily activity alone (see Goffman E, *Behavior in Public Places*. New York; Free Press, 1963, p.13-14)

For peer review only - http://bmjopen.bmj.com/site/about/guidelines.xhtml

Survivor (1.06): "A woman turned up … and she said, Are you OK? I said, I'm fine, thank you … And she said, Well you don't seem fine. I said, I'm just looking at the view. I was angry that she was meddling. It wasn't her business. I was perfectly fine on my own. She said, Mmm, do you want to climb back over and we can both look at the view? And I said, No."

Other interveners were more assertive in their approach, using some degree of verbal or physical restraint:

Survivor (1.07): "A woman came over … She put her hand on my arm … and she said, You don't need to do this. It's OK, nothing's that bad."

Having approached the person and made an opening gambit, interveners then found themselves committed to remaining present for as long as it took, either to accomplish a handover to emergency services or to be sure that the person was no longer a risk to themselves. In some cases this took several hours. During that time, they largely improvised, trusting their instincts, drawing on their personal resources, and trying to show warmth and compassion in whatever way they could. The following elements emerged as important in the accounts of both groups.

Exchanging names: Names were not always exchanged, but when they were this was seen as having played an important part in "bursting the bubble" and personalising the encounter. By giving their name, interveners demonstrated that they were authentic or "real", which could have an anchoring effect:

For peer review only

Survivor (1.06): "I remember her name. It's the one thing that I kept saying to myself ... It was something to hold onto, more tangibly than like a physical thing, I think."

Asking simple, factual questions: Interveners went on to ask simple questions in the usual manner of 'getting to know you', enquiring where the person lived, whether they worked or were studying, and so on. This served multiple purposes, beyond establishing the facts. It gave the suicidal person something to focus on, drawing their attention away from imminent death and helping them to reconnect with themselves and their normal world. It also served gradually to build rapport between suicidal person and intervener. One participant highlighted the importance of persisting, if at first the suicidal person is unable to answer:

Survivor (1.01): "It did take quite a lot of asking ... She kept repeating the same questions ... When she asked them the first time I wasn't in a place where I could answer, but as I sort of came back into reality a bit, rather than the world inside my head ... You start to feel a bit less disconnected ... almost like it's foggy and then the fog lifts a bit, enough for you to see that there is something beyond the fog, like if the fog is suicide."

Interveners also enquired about family members and friends, and if there was someone who could be contacted (see below, *Summoning help)*.

Sharing lived experience and personal stories: As in any normal conversation, interveners reciprocated by sharing information about themselves. When interveners had their own lived experience of mental health problems, participants in both groups believed it was

For peer review only - http://bmjopen.bmj.com/site/about/guidelines.xhtml

useful to share this in order to establish credibility and trust and to provide hope of recovery. At the same time, interveners were careful to avoid focusing attention on themselves or presuming to know what the suicidal person was going through.

"Keeping it light", and not asking why: Participants in both groups expressed a strong belief that this was not the time or place to probe the reasons for wanting to die. Those in the survivor group who had been asked what had brought them to the point of suicide reported that they had been unable to talk about it, whilst others said they were glad they had not been asked. As they emerged from the 'bubble', their predominant feelings were those of ambivalence about being prevented from carrying out their plan, embarrassment and exhaustion, and their immediate need was simply for the comforting presence of another human being, not an intellectually demanding 'talking therapy' session that kept them focused on the distress:

> *Survivor (1.06): "She started saying, What's happened? Why do you feel like this? And I didn't say anything. I couldn't give her an answer. It was too complicated."*

> *Survivor (1.11): "She didn't ask why… I don't think I would have been able to answer her anyway. I would have probably bolted."*

Some interveners did ask what had brought the person to this point and some, but not all, received a response. Others reported that they had shied away from "getting in too deep" or sensed that it was not appropriate to do so, believing that exploring the precipitating events or stressors could wait until later and that making the person dwell on their problems at this point would not be useful:

For peer review only - http://bmjopen.bmj.com/site/about/guidelines.xhtml

Intervener (2.13): "I realised I didn't need to go into the why, didn't need to establish any of that stuff."

Nor was it considered helpful for a stranger to present a suicidal person with reasons for living. This was likely to be regarded as presumptuous and to provoke anger, especially when the intervener lacked lived experience of depression or suicidality.

Instead of dwelling on distress, interveners tried to keep the conversation "light" and focused on normal, everyday things, a number of them admitting that they were simply trying to buy time or alleviate their own discomfort. Survivors affirmed that this was nonetheless an effective way of "holding" them:

Intervener (2.13): "It was quite a random conversation ... I don't like silence so I just filled it with things that popped into my head."

Survivor (1.05): "He just started rambling on about his own life, which was kind of lovely. Like he was just keeping me listening even if I wasn't talking ... I could still relate to him in a way. He was saying he'd just come back from a party or something…"

Silences, and just being present: Other interveners recognised that it was possible to connect with a person without words and that silence could provide the suicidal person with a welcome respite from the intrusive and negative babble that was going on inside their own head:

For peer review only - http://bmjopen.bmj.com/site/about/guidelines.xhtml

Survivor (1.03): "She didn't really talk too much. She was just there … And she stayed with me and bought me another cup of coffee … and eventually the voices were stopping and I felt like I was in the moment."

The mere presence of another person was also a powerful deterrent. Survivors consistently reported that the arrival of another person made it impossible to go through with their planned suicidal act. Even at the point of crisis, they still seemed able to think of others:

Survivor (1.08): "I wanted them to just go away, but they didn't … They were there and I didn't want to do anything in front of their little baby … because he was old enough to know what had happened."

Embodied communication or body language was as powerful as words in establishing a non-threatening presence and conveying warmth and empathy, and included the following elements.

Proximity and positioning of self: Interveners described how they positioned themselves vis-à-vis the suicidal person, placing themselves on an equal plane (e.g. sitting or squatting) and in the same orientation (alongside and facing the same way) so as to appear friendly rather than confrontational. Maintaining a respectful distance was seen as important by those who were suicidal:

Survivor (1.09): "I remember saying, Don't come so close. I was really kind of adamant about that because this was my thing and my space."

For peer review only - http://bmjopen.bmj.com/site/about/guidelines.xhtml

Caring gestures and touch: Interveners attended to the suicidal person's physical needs, offering them a coat, a cigarette or a warm drink. Participants in both groups recognised that the warm touch of another human being could be very powerful in breaking the isolation of the suicidal person, but several survivors reported that histories of abuse had left them with a horror of physical contact and stressed the importance of asking permission before touching:

Survivor (1.03): "She said, Do you mind if I touch your hand? She asked me, and I can remember feeling the touch of a human person so clearly."

Survivor (1.06): "Her hand was on my arm and it was warm ... I was this frozen ice person, who had given up on everything, and she'd come over with sort of warm words and this physically warm touch that I didn't necessarily want to pull away from."

General demeanour: Survivors consistently reported that the manner in which the intervener spoke to them was as important as the words spoken, if not more so. Calmness (not showing alarm), authenticity (just being themselves, being "real"), steadfastness ("not budging") and sincerity were traits that were mentioned again and again and that had the effect of making the person feel safe, valued and connected. The mere act of stopping and saying something gave a powerful signal to the person, at their lowest point, that they mattered:

Survivor (1.07): "The fact that you do stop makes all the difference, rather than walking past ... The fact that [on three separate occasions] people stayed and talked to me ... It

For peer review only - http://bmjopen.bmj.com/site/about/guidelines.xhtml

was like, right now you matter and your life matters and we care what happens to you. And that was a genuine thing."

ii) Moving to a safer location

Nearly all interveners had to work out how to move the person away from the means of suicide to a safer location. Efforts to do so took one of the following three forms.

Forcible restraint: Some interventions began with a physical act of 'grabbing', designed to effect the swift and forcible removal of a person from a situation of extreme danger, before any attempt was made to engage in verbal rescue work. Whilst no-one regarded this as ideal, some survivors recognised that it had been the right thing to do in the circumstances:

Survivor (1.01): "I remember hearing two people coming up the steps … and one of them just pulled me backwards off the ledge … Had I not been pulled down, I doubt I would have engaged at all because I was so inside of myself."

The risks involved in grabbing were also clearly illustrated. In one case, two women walking on a riverside path saw a person ahead of them on the wrong side of a bridge, leaning out over the water and in extreme peril. Having reached the person in time and grabbed both her arms, they then found themselves trapped, lacking the strength to pull her back over the railing, struggling to keep hold, and able neither to engage her in conversation nor to attract the attention of passing motorists. The situation was eventually resolved, but not without injury to one of the interveners.

For peer review only - http://bmjopen.bmj.com/site/about/guidelines.xhtml

Grabbing was also seen as a purely instrumental action, which, whilst it may have been effective in saving the life on that occasion, did nothing to contribute to longer-term recovery. Interventions at the other end of the control/agency spectrum (Figure 2), which encouraged self-rescue, were deemed more likely to have an ongoing positive and protective effect.

Direct appeal ("Please come away from the edge"): This was an option when there was less urgency and it was believed that the person had the capacity to respond. Some approached it in an oblique manner:

> *Intervener (2.21):"I didn't want to confront her and say, Don't jump! … I said, Do you realise the cliff edge is really crumbly there? I'm quite worried that it might collapse. Why don't you step back a bit?"*

Indirect ("Let's go somewhere for a coffee"): Offering to buy the person a warm drink and something to eat performed multiple purposes. It could be both a simple act of kindness and validation, and also a ploy to move the person to a safer location. Either way, it was a highly effective action, serving to ease the person back into the normal, everyday world (Figure 1):

> *Intervener (2.12): "I was trying to work out how I could get her out of the tube [underground rail network] and somewhere safer … So I said, Let's go and get a cup of tea somewhere and have some cookies … And she helped herself to a cookie and there were signs she was coming to a little bit … and she sort of opened up to me then."*

For peer review only - http://bmjopen.bmj.com/site/about/guidelines.xhtml

iii) Summoning help

There were three potential sources of support on which interveners could call: a family member or friend nominated by the suicidal person; other passers-by, and the emergency services.

Family member or friend: Interveners typically asked about family and friends as part of "bursting the bubble," but it was also a way to identify someone who might come and take the person home. The following example shows how a brief verbal exchange performed both tasks at once. The intervener appears initially to be trying to establish if there is someone who can be called to provide support. The suicidal person can think of no-one, but as the exchange proceeds she gradually remembers her extended family and the closeness of her social bonds (Figure 1). The sight of the photograph on her phone represented a turning point, after which she could no longer go through with the act of suicide and agreed to contact one of her daughters:

> *Survivor (1.03): "She said, Is there somebody you can ring? And I said, No, there's nobody. And she said, Have you got any children? And I said, Yes, I've got two daughters. Oh, have you got a picture, she said. And I got out my phone and showed her a picture of my six grandchildren…"*

Other passers-by: Efforts to enlist help from other passers-by were rarely successful. Participants in both groups gave accounts of busy locations and people passing by, either not noticing or pretending not to notice what was happening. Several interveners made repeated and desperate attempts to signal to other passers-by but were ignored, although in some cases it transpired that a member of the passing crowd had taken it upon

For peer review only - http://bmjopen.bmj.com/site/about/guidelines.xhtml

themselves to call emergency services. However, other interveners rejected offers of help, fearing that the presence of another person would threaten the one-to-one connection and trust they were establishing with the suicidal person.

Emergency services: Interventions typically ended with the arrival of emergency services. Rail staff and highways officers had usually alerted emergency services prior to approaching the suicidal person, so that their task was simply to keep the person safe until specialist help arrived. For members of the public it was much more challenging. None of them had thought to call emergency services prior to intervening, and once they had approached and embarked on rescue work it became very difficult to do so. They feared that any move to do so, in particular any mention of police involvement, would trigger panic and flight. In some cases, the intervener asked permission to call for help and it was refused, leaving them alone and floundering. Others resorted to subterfuge or were fortunate that a member of the crowd called for help on their behalf, often unbeknown to them. Several interveners reported being unsure which service to ask for. The following example illustrates the multi-tasking nature of intervention work, the fragility of the connection with the suicidal person, and the impossibility of maintaining it whilst doing other things:

> *Intervener (2.12): "I was trying to get my phone out of my handbag without losing eye contact ... And I glanced down to unlock it and she ran ... In that split second I lost her ... So I was going after her and I was calling 999 as well, and I remember on the phone feeling quite confused because I really wasn't sure what service to be asking for ... I said, There's a lady running into the road, and they said, Well, what service do you need? And I said, I don't know..."*

For peer review only - http://bmjopen.bmj.com/site/about/guidelines.xhtml

Interveners who had worked hard to build an emotional connection with the person expressed misgivings about medicalising the situation by calling an ambulance or criminalising it by involving the police.

Endings and aftermath

Ending an intervention without a handover to emergency services or to a family member or friend was fraught with difficulty. Interveners described being desperate to extricate themselves, sometimes after several hours, but not knowing whether it was safe to let the person to go on their way and sometimes debating whether they should invite the person home with them.

Endings involving a handover were not necessarily any easier. Interveners experienced intense relief as they themselves were 'rescued', but were often left with feelings of exclusion, loss and fear of consequences for the person (especially when they were taken away in handcuffs or sectioned under the Mental Health Act). Some were left feeling disturbed by what had happened. Those who had been on their way to work at the time described the surrealness of trying to carry on *as normal* after the extraordinariness and intensity of the encounter.

For peer review only - http://bmjopen.bmj.com/site/about/guidelines.xhtml

DISCUSSION

This is the first empirical study to examine the role of passing strangers in preventing suicides. Intervention was seen to involve three main tasks: "bursting the bubble" (reconnecting with self, others and everyday world); moving to a safer location, and summoning help. Interveners accomplished these tasks in a range of ways, using both verbal and non-verbal communication and different degrees of restraint.

Those who had been suicidal (the survivor group) typically described themselves as being dissociated or "in a bubble," cut off from themselves and everything around them. Dissociative states have long been linked with suicide.[19] Orbach postulates that detachment from the body, indifference to physical pain and lack of affect are necessary in order for an individual to go through with a suicidal act. Absence of emotion is a subtle and counterintuitive clue. In this regard, suicide is like drowning insofar as the person does not look like we expect them to. A drowning person is not thrashing about and shouting for help, as commonly portrayed in film; they appear silent and still and are easily overlooked. Likewise the suicidal person rarely conforms to the stereotypical image of the anguished soul, as depicted in Munch's painting of The Scream. This is an important message to convey to the public. In an online survey of rail staff, Mackenzie and colleagues found that a high proportion of staff expected suicidal individuals to exhibit visible signs of distress,[20] suggesting that this may be a common misperception.

With few outward clues except location, interveners relied on their own 'sixth sense' that something was wrong. Some quality of oddness or incongruity triggered an alarm inside them, which they were able to hear. Thus recognition relies as much on an *internal* state

For peer review only - http://bmjopen.bmj.com/site/about/guidelines.xhtml

of openness, curiosity, attunement to others and attention to one's own inner voice as on any *external* signs.

Openness needs to be combined with readiness for action. The interveners in our study were ordinary people; they did not possess any special knowledge or skills. What was unusual about them was their lack of hesitancy and their disregard of the social risks involved in approaching a stranger in a public place. Most people are kept from doing so by fear of a rude rebuff and resulting embarrassment.[21] The interveners we interviewed were not fearless but, on the principle of "Feel the fear and do it anyway", they did it anyway. They also showed themselves to be resourceful, able to dig around in their personal "experience bag" and find things that worked, without necessarily knowing how or why.

Our findings suggest that people do not need to be provided with an intervention script, nor should they be afraid of saying "the wrong thing." The words the intervener spoke were not nearly as important as how they made the person feel. Survivors described interveners as having broken through the dissociative state, dispelling feelings of fear, isolation and worthlessness and making them feel safe, validated and re-connected. The basic mechanisms are depicted in Figure 3.

[INSERT FIGURE 3]

This model of effective intervention, with feeling rather than reasoning at its core, runs counter to conventional wisdom, as contained in programmes such as Applied Suicide Intervention Skills Training (ASIST)[22] and in academic literature.[8] The ASIST model

For peer review only - http://bmjopen.bmj.com/site/about/guidelines.xhtml

stresses the importance of listening closely to the person's reasons for dying and working with them to uncover reasons for living. Its focus is on verbal reasoning. Likewise, Omer and Elitzur's proposed script for a last-minute crisis intervention insists on challenging suicidal thinking. The authors compare the situation to a court of law in which the defence lawyer (the intervener) picks holes in the prosecutor's (the suicidal person's) story in an attempt to win the argument.[8] Our interveners were not attempting to win a legal battle. Nor were they trying to imitate therapists or crisis negotiators. They were just being themselves and reaching out as one human being to another. This is not to say that reasoning and verbal persuasion played no part, or that they may not be important at other stages of the suicidal process, but few of our survivors reported having been swayed by logic or argument. What they recalled most strongly about those who had helped them was their authenticity and their compassion. What made the difference was the simple fact that someone cared enough not to walk on by.

Our model may not be appropriate to other settings. A situation involving an unknown person on the point of carrying out a suicide plan in a public location is very different from other crisis settings. These include domestic or social settings, where a personal relationship exists between the two parties;[15] clinical settings, where a professional relationship and statutory responsibility for risk management exist,[23-25] and telephone counselling services, where the suicidal person has invited a talking intervention by calling a crisis line.[26 27] In these situations, the person may not yet be at the stage having formulated a suicide plan. Our study suggests that rather than there being a 'one size fits all' model of human intervention, as the ASIST training supposes, different social contexts and stages of the suicidal process may require different approaches.

For peer review only - http://bmjopen.bmj.com/site/about/guidelines.xhtml

Our findings show clearly that anyone who wants to intervene can safely do so. Last-minute intervention requires no specific learning and can be highly effective when spontaneous and unscripted. In 2018, Network Rail in partnership with Samaritans launched a campaign entitled "Small Talk Saves Lives", encouraging rail travellers simply to say 'Hello' and strike up a normal conversation if they are concerned about someone.[28] Our data confirm that this campaign message is entirely appropriate as far as the initial approach to a vulnerable person is concerned. However, it fails to recognise the intense, prolonged and taxing nature of intervention, the complex juggling acts that interveners may have to perform in the course of trying to keep someone safe, and the troubling emotions they may be left with. It is no small thing to save a life. The conundrum for public education is how to prepare people adequately for the challenges they may face without deterring them from intervening.

A set of simple public education messages is needed to encourage people to recognise and reach out to those who may be at risk of suicide in public locations. We are working with a wide range of non-academic partners to develop and disseminate these.

FIGURE LEGENDS

Figure 1: Main intervention tasks, components and interconnections

Figure 2: Control/agency spectrum

Figure 3: Proposed mechanisms of action in an effective intervention by a stranger in a public location

For peer review only - http://bmjopen.bmj.com/site/about/guidelines.xhtml

ACKNOWLEDGEMENTS

We thank the participants in both groups for sharing their stories. We are also grateful to Network Rail and Highways England for help with recruiting interveners, to Jonny Benjamin MBE, Neil Laybourn (the original *'Stranger on the Bridge'*), and the other members of our expert advisory group.

AUTHOR CONTRIBUTIONS

CO had the idea for the study and conducted public involvement. She designed the study with CA, managed the project, led the analysis and interpretation of data and wrote the manuscript. JD collected data and analysed the transcripts. Both JD and CA commented on emerging drafts of the manuscript. All authors have read and approved the final version. CO is the guarantor.

FUNDING STATEMENT

This work was supported by the Medical Research Council grant number MR/P01707X/1.

COMPETING INTERESTS

The authors declare that they have no competing interests.

ETHICS APPROVAL

Ethical approval was granted by the University of Exeter Medical School Research Ethics Committee.

For peer review only

DATA AVAILABILITY STATEMENT

We do not have consent from participants to make their transcripts publicly available. Reasonable requests for anonymised primary data for the purposes of checking the accuracy of our findings and interpretation may be sent to the corresponding author: c.v.owens@exeter.ac.uk

REFERENCES

1. Owens C, Lloyd-Tomlins S, Emmens T, et al. Suicides in public places: findings from one English county. *European Journal of Public Health* 2009;19(6):580-2.
2. Channel 4. http://www.channel4.com/programmes/the-stranger-on-the-bridge 2015 [Accessed 07.06.2019]
3. Department of Health. Preventing suicide in England: A cross-government outcomes strategy to save lives. London: HM Government 2012
4. Owens C, Hardwick R, Charles N, et al. Preventing suicides in public places: A practice resource London: Public Health England 2015. https://www.gov.uk/government/publications/suicide-prevention-suicides-in-public-places.
5. Cox G, Owens C, Robinson J, et al. Interventions to reduce suicides at suicide hotspots: A systematic review. *BMC Public Health* 2013;9(13):214.
6. Pirkis J, Spittal M, Cox G, et al. The effectiveness of structural interventions at suicide hotspots: a meta-analysis. *Int J Epidemiol* 2013;42(2):541-8.
7. Pirkis J, Too L, Spittal M, et al. Interventions to reduce suicides at suicide hotspots: a systematic review and meta-analysis. *The Lancet Psychiatry* 2015;2(11):994-1001.
8. Omer H, Elitzur A. What would you say to the person on the roof? A suicide prevention text. *Suicide Life Threat Behav* 2001;31(2):129-39.

For peer review only - http://bmjopen.bmj.com/site/about/guidelines.xhtml

9. World Health Organization. Preventing Suicide: A Resource for Police, Firefighters and Other First Line Responders. Geneva: World Health Organization 2009. http://www.who.int/mental_health/prevention/suicide/resource_firstresponders.pdf [Accessed 07.06.2019]

10. Lanceley F. On-Scene Guide for Crisis Negotiators (2nd edition). Boca Raton, Fl: CRC Press 2003.

11. Greenstone J. Teen Suicide: Negotiating with the Young Suicidal Actor. *Journal of Police Crisis Negotiations* 2008;3(2):61-64. doi: 10.1300/J173v03n02_07

12. Fowler W, Maguire J. Dealing with Defenestrators: Immediate Interventions. *Journal of Police Crisis Negotiations* 2001;1(2):41-51.

13. Holloway W, Jefferson T. Doing Qualitative Research Differently: Free Association, Narrative and the Interview Method. London: Sage 2000.

14. Wengraf T. Qualitative Research Interviewing: Biographic Narrative and Semi-Structured Methods. London: Sage 2001.

15. Owens C, Owen G, Belam J, et al. Recognising and responding to a suicidal crisis in the family and social network: qualitative study *BMJ* 2011;343:d5801.

16. Cannon S. Social research in stressful settings: difficulties for the sociologist studying the treatment of breast cancer. *Sociol Health Illn* 1989;11(1):62-77.

17. Lee RM. Doing research on sensitive topics. London: Sage 1993.

18. Holstein K, Gubrium J. Active interviewing. In: Silverman D, ed. Qualitative Research: Theory, Method and Practice. London: Sage 1997.

19. Orbach I. Dissociation, physical pain and suicide: A hypothesis. *Suicide and Life-Threatening Behavior* 1994;24(1):68-79.

For peer review only - http://bmjopen.bmj.com/site/about/guidelines.xhtml

20. Mackenzie J, Borrill J, Hawkins E, et al. Behaviours preceding suicides at railway and underground locations: a multimethodological qualitative approach. *BMJ Open* 2018;8:e021076. doi: doi: 10.1136/bmjopen-2017-021076

21. Goffman E. Behavior in Public Places: Notes on the Social Organization of Gatherings. New York: Free Press 1963.

22. LivingWorks Education. https://www.livingworks.net/programs/asist/ 2014-16 [Accessed 07.06.2019] [

23. Appleby L, Morriss R, Gask L, et al. An educational intervention for front-line health professionals in the assessment and management of suicidal patients (The STORM Project). *Psychological Medicine* 2000;30(4):805-12.

24. Hendin H, Maltsberger J, Lipschitz A, et al. Recognizing and responding to a suicide crisis. *Suicide Life Threat Behav* 2001;31(2):115-28.

25. Department of Health. Best Practice in Managing Risk: Principles and Evidence for Best Practice in the Assessment and Management of Risk to Self and Others in Mental Health Services. London: Department of Health 2009. https://assets.publishing.service.gov.uk/government/uploads/system/uploads/attachment_data/file/478595/best-practice-managing-risk-cover-webtagged.pdf [Accessed 07.06.2019].

26. Mishara B, Chagnon F, Daigle M, et al. Which helper behaviors and intervention styles are related to better short-term outcomes in telephone crisis intervention? Results from a Silent Monitoring Study of Calls to the U.S. 1-800-SUICIDE Network. *Suicide Life Threat Behav* 2007;37(3):308-21.

27. Gould M, Cross W, Pisani A, et al. Impact of Applied Suicide Intervention Skills Training on the National Suicide Prevention Lifeline. *Suicide and Life Threatening Behavior* 2013;43(6):676-91. doi: 10.1111/sltb.12049

For peer review only - http://bmjopen.bmj.com/site/about/guidelines.xhtml

28. Samaritans. https://www.samaritans.org/media-centre/our-campaigns/small-talk-saves-lives 2018. [Accessed 07.06.2019]

For peer review only

For peer review only - http://bmjopen.bmj.com/site/about/guidelines.xhtml

Figure 1: Main intervention tasks, components and interconnections

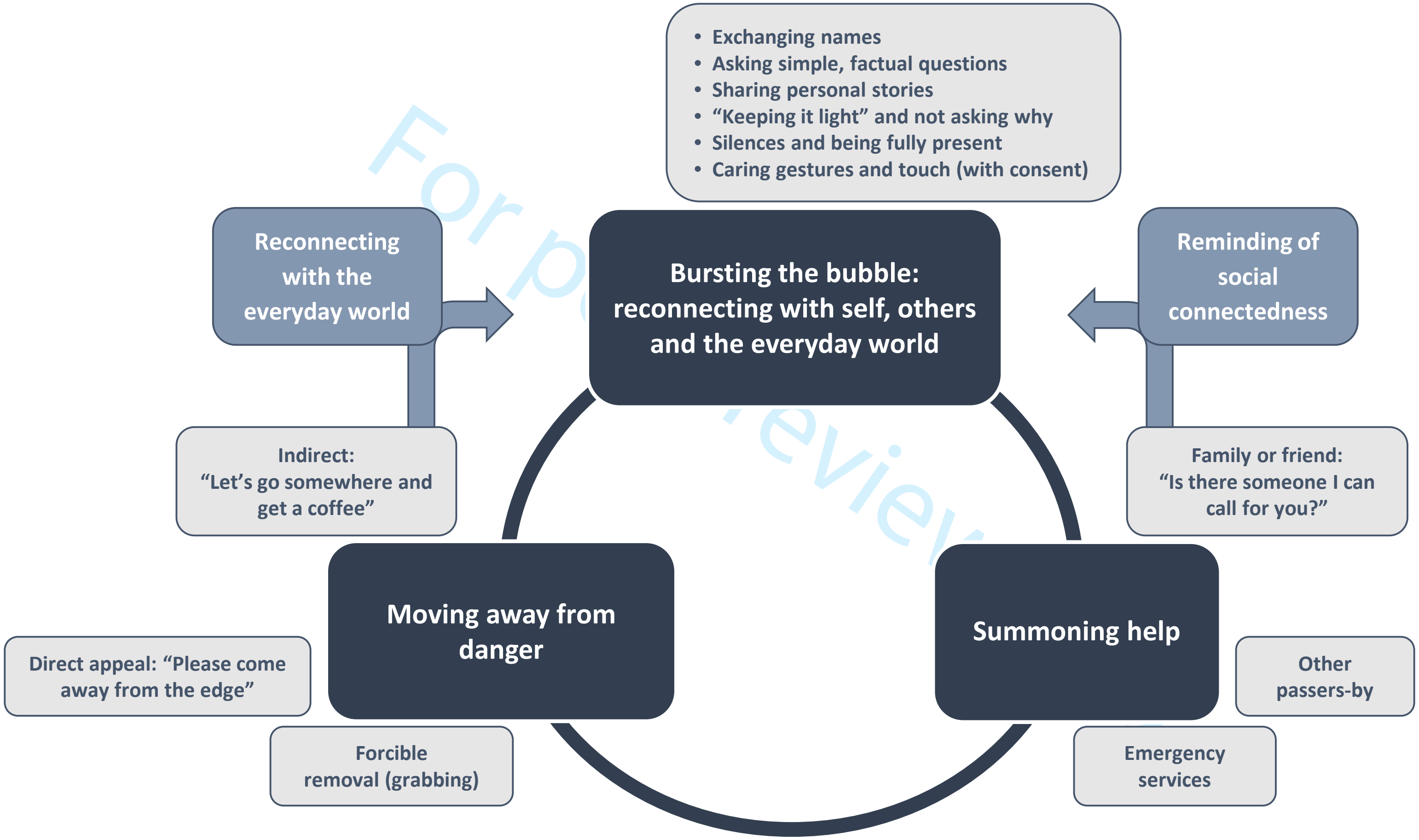

Figure 2: Control/agency spectrum

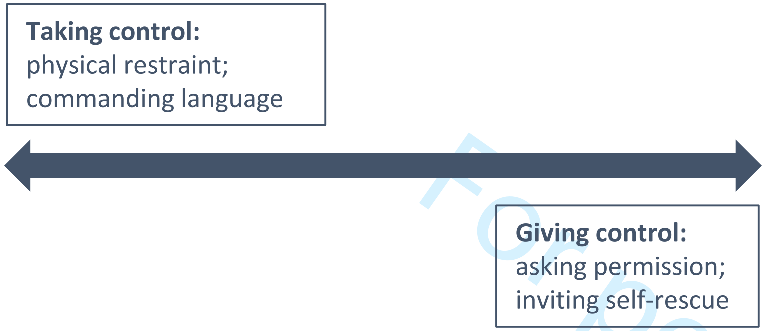

For peer review only

Figure 3: Proposed mechanisms of action in an effective intervention by a stranger in a public location

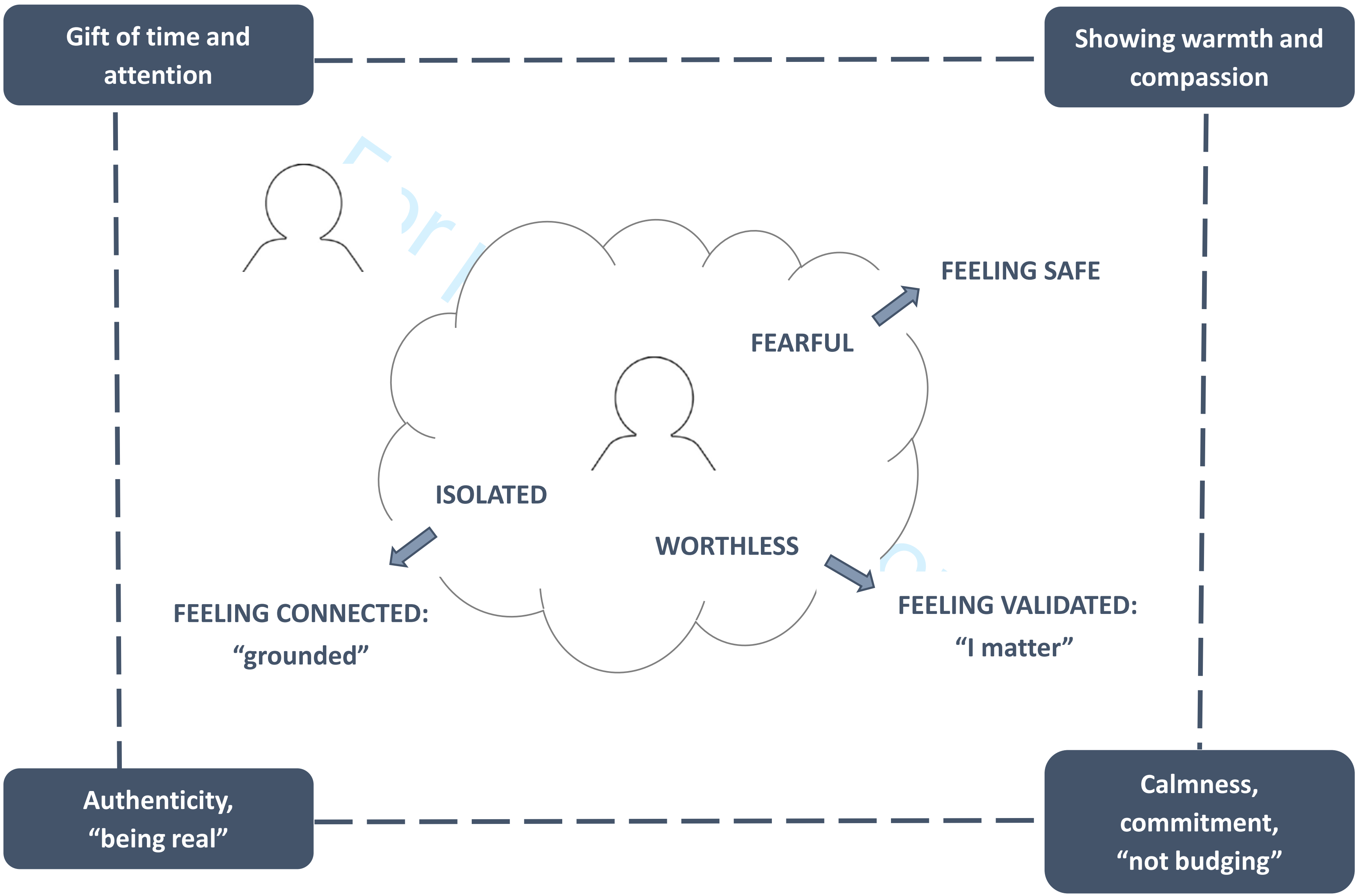

Topic guide
Group 1: Those with experience of being stopped from taking their own lives

Checklist:
- Information sheet and consent form
- Tape recorder
- Spare batteries
- Biscuits or cake
- Turn phone to silent

Preamble:
- *Thanks for coming forward; importance of understanding the situation from your point of view*
- *Brief recap of aims of study*
- *Recap of confidentiality*
- ***Ask directly** about current mental state and suicidality: "Have you had any thoughts of suicide recently [in the last week or two]? How are you feeling right now?" If currently receiving psychiatric care, do they have a letter from psychiatrist? If in any doubt, advise not to proceed.*
- *Stopping rule: "I realise that talking about that time will be difficult for you. You are completely free to stop anytime you wish, or you can take a break and then decide whether or not to carry on. Please tell me if talking about it triggers thoughts of harming yourself or makes you feel in any way unsafe. If that happens, you **must** stop."*
- *Sign consent form*
- *Brief outline of interview*

Opening free narrative:

"So I know that you came close to taking your own life in [month/year]. In your own time, please tell me what led up to it and what happened on that particular day."

Prompts and suggested follow-up. Ensure the following are fully explored:

Setting:
- Clarify where exactly it took place
- Why there (choice of location)? Why that method of suicide? Had you thought about other places and possibly other methods of ending your life? How carefully planned was it?
- How did you get there? How long did the journey take?
- What was going through your mind when you got there? [Probe: thinking and feeling]

Intent:
- How much did you want to die?
  or
  Did you think someone might try to stop you? Was any part of you hoping that might happen?

Intervention:
- Who approached you and how?
- What was the first thing they said to you? Then what…?
- How did they say it? [Probe: body language, positioning, eye contact, touch, other non-verbal signals, pace and timing, general manner and tone of voice.
- How did it make you feel?

For peer review only

- What other actions did they take, including calling emergency services?
- What helped most, made the most difference?
- What wasn't helpful, irritated or angered you?
- What sort of things should people avoid saying or doing?

- What message would you like to give to anyone who is unsure about whether or not to intervene?

- Thinking more broadly about prevention and about that particular place, apart from someone stepping in and talking to you, is there anything else that might have prevented you from going through with the suicide? [Prompt if necessary: e.g. higher parapet on bridge, fencing on railways, emergency telephone…]

Participant's background

- Age
- Ethnicity
- Region (London; South East; South West etc)
- Employment status [employed; unemployed; student; homemaker; carer; retired…]
- If working, what do they do?

- Have you kept in touch with the person who helped you? If so, how would you feel about us possibly contacting them and asking them if they would be interviewed?

- Is there anything else you'd like to add?

Close:

- *Thanks again etc.*
- *Offer Amazon voucher and check email address*
- *Tell them they can get in touch if they think of anything afterwards that they'd like to add or change*
- *Ask if they'd like to receive further information about the study and a summary of the findings*
- *Ask if they know others who've been in similar situation who might want to take part*
- *Ask if they have any ideas about how to advertise study.*

Debrief/safety check:

- *How are they feeling right now? **Ask directly**: "Has talking about that day made you feel like harming yourself again?"*
- *Do they have plans for the rest of the day? Where are they planning to go [immediately after the interview]; are they meeting up with anyone? Offer to stay with them until then, or ask them to text you when they get there*
- *Give details of organisations to contact for support if they become upset later*
- *If they give any cause for concern, ask if you can accompany them to a place of safety (a trusted friend's house or A&E).*

For peer review only

Topic guide
Group 2: Those with experience of intervening

Checklist:

- Consent form
- Tape recorder
- Spare batteries
- Biscuits or cake
- Turn phone to silent

Preamble:

- *Thanks for coming forward; importance of understanding the situation from your point of view*
- *Brief recap of aims of study*
- *Recap of confidentiality*
- *Possible that it may be upsetting to recall what happened. If that happens, please tell me and we will stop the interview. You are completely free stop anytime you wish, or you can take a break and then decide whether or not to carry on.*
- *Sign consent form*
- *Brief outline of interview*

Opening free narrative:

"So I know that in [month/year] you saw someone in a public place who you thought was at risk of harming themselves and that you tried to help them. In your own time, please tell me what happened on that particular day."

Prompts and suggested follow-up. Ensure the following are fully explored:

Setting

- Clarify where exactly it took place
- What exactly were you doing when you first noticed the person? Where were you going? Paint a picture of the scene.

Recognition

- What was the first thing you noticed about them?
- How quickly did you realise that they might be in need of help of some sort?
- Did you immediately recognise that they were at risk of harming themselves?
- What was it that enabled you to know that? [Probe: features, appearance, behaviour of suicidal person **plus** participant's own background /previous experience or exposure]

Preparedness

- What went through your mind when you realised that?
- How quickly did you decide to approach them, and how did you make that decision?
- Did you know what to say or do, or were you just 'winging it'?
- How confident did you feel that you could help them? [Probe for: fears about intervening; feelings about competence]

For peer review only

Intervention

- What was the first thing you said or did?
- Then what…?
- What was going through your mind while it was all happening? How were you feeling?
- Do you think you made any mistakes? Anything you regret saying or doing? If so, what?
- What would you do differently another time?
- Do you have any thoughts about what was most helpful?

Personal consequences:

- What impact has it had on you? [Probe: immediate; longer-term, depending on how long ago; future]
- Would you do it again? Why/why not?
- What message would you give to anyone who is unsure about whether or not to intervene in a situation like the one you encountered?

Training

- If not already mentioned: Have you received any mental health or suicide prevention training? If so, what and when?
- Do you think members of the public should receive basic suicide prevention training, in the same way that they get first aid training? What form do you think it should take?
- What sort of information would have been helpful to you in the situation you found yourself in?

Participant's background

- Age
- Ethnicity
- Region (London; South East; South West etc)
- Employment status [employed; unemployed; student; homemaker; carer; retired…]
- If working, what do they do?

- Have you kept in touch with the person you helped? If so, how would you feel about us possibly contacting them and asking them if they would be interviewed?

- Is there anything else you'd like to add?

Close:

- *Thanks again etc.*
- *Offer Amazon voucher and check email address*
- *Tell them they can get in touch if they think of anything afterwards that they'd like to add or change*
- *Ask if they'd like to receive further information about the study and a summary of the findings*
- *Ask if they have any links with others in similar situation*
- *Ask if they have any ideas about how to advertise the study.*

Safety check:

- *How are they feeling right now? Has it been upsetting recalling the events of that day?*
- *If so, check plans for the rest of the day? Do they have someone they can meet up with?*
- *Give details of organisations to contact for support*

For peer review only

Consolidated criteria for reporting qualitative studies (COREQ): 32-item checklist

**Guide questions/description**	**Authors' response**
Domain 1: Research team and reflexivity	
Personal Characteristics<br>1. Interviewer/facilitator. Which author/s conducted the interview ~~or focus group~~?	Reported in manuscript, p.8 (all page numbers refer to revised manuscript)
2. Credentials. What were the researcher's credentials? E.g. PhD, MD	Master's degree and PhD
3. Occupation. What was their occupation at the time of the study?	University Research Fellow
4. Gender. Was the researcher male or female?	Female
5. Experience and training. What experience or training did the researcher have?	Extensive track record in use of qualitative research methods, including PhD and 10 years' post-doctoral experience. Reported on p.8
Relationship with participants<br>6. Relationship established. Was a relationship established prior to study commencement?	Email and telephone contact prior to interview
7. Participant knowledge of the interviewer. What did the participants know about the researcher? e.g. personal goals, reasons for doing the research	The interviewer shared relevant personal information during preliminary conversations.
8. Interviewer characteristics. What characteristics were reported about the interviewer/facilitator? e.g. Bias, assumptions, reasons and interests in the research topic	Insufficient space to report
Domain 2: study design	
Theoretical framework<br>9. Methodological orientation and Theory. What methodological orientation was stated to underpin the study? e.g. grounded theory, discourse analysis, ethnography, phenomenology, content analysis	Inductive thematic analysis, described in detail on p.8-9
Participant selection<br>10. Sampling. How were participants selected? e.g. purposive, convenience, consecutive, snowball	There are a limited number of people in the general population who meet the inclusion criteria and they are impossible to identify except by advertising the study and hoping they will come forward. This was the only possible sampling strategy.
11. Method of approach. How were participants approached? e.g. face-to-face, telephone, mail, email	Potential participants expressed interest by emailing a project mailbox. They were then contacted by return email, with a participant information leaflet attached, and were invited to receive a telephone call to discuss the study in more detail and ask questions. Once the researcher was confident that they were eligible to participate and happy to proceed, interview

	arrangements were made. Written consent was taken on the day of interview.
12. Sample size. How many participants were in the study?	Reported in manuscript, p.10
13. Non-participation. How many people refused to participate or dropped out? Reasons?	n/a
Setting<br>14. Setting of data collection. Where was the data collected? e.g. home, clinic, workplace	Reported in manuscript, p.8
15. Presence of non-participants. Was anyone else present besides the participants and researchers?	No
16. Description of sample. What are the important characteristics of the sample? e.g. demographic data, date	Reported in manuscript, p.10 and Table 1
Data collection<br>17. Interview guide. Were questions, prompts, guides provided by the authors? Was it pilot tested?	Reported in manuscript, p.8. Interview guides uploaded as supplementary files.
18. Repeat interviews. Were repeat interviews carried out? If yes, how many?	No repeat interviews
19. Audio/visual recording. Did the research use audio or visual recording to collect the data?	Reported in manuscript, p.8
20. Field notes. Were field notes made during and/or after the interview ~~or focus group~~?	Yes
21. Duration. What was the duration of the interviews ~~or focus group~~?	Reported in manuscript, p.8
22. Data saturation. Was data saturation discussed?	Reported in manuscript, p.7
23. Transcripts returned. Were transcripts returned to participants for comment and/or correction?	No. It was not considered ethical for the 'survivor' group to read accounts of their own past suicidal crises without a researcher present to offer support should they become distressed or find the accounts triggering.
Domain 3: analysis and findings	
Data analysis<br>24. Number of data coders. How many data coders coded the data?	One researcher (JD) coded all transcripts using NVivo; another researcher (CO) coded independently using non-computerised methods and also checked JD's NVivo coding of several full transcripts.
25. Description of the coding tree. Did authors provide a description of the coding tree?	Available from the authors
26. Derivation of themes. Were themes identified in advance or derived from the data?	Reported in manuscript, p.8-9
27. Software. What software, if applicable, was used to manage the data?	Reported in manuscript, p.9
28. Participant checking. Did participants provide feedback on the findings?	Yes. We presented findings on three occasions to an expert advisory group comprising people with lived experience and a range of external stakeholders. We also

	sent a summary of findings to all participants and invited their comments. Reported in manuscript, p.9
Reporting<br>29. Quotations presented. Were participant quotations presented to illustrate the themes / findings? Was each quotation identified? e.g. participant number	Yes to both questions. Extensive illustrative quotations are presented. These were identified by participant group (intervener or survivor) and individual number.
30. Data and findings consistent. Was there consistency between the data presented and the findings?	Yes, we would hope so.
31. Clarity of major themes. Were major themes clearly presented in the findings?	Yes, we would hope so. Major themes are listed on p.11
32. Clarity of minor themes. Is there a description of diverse cases or discussion of minor themes?	Yes, sub-themes are described on pp.15-26. Major and minor themes are also represented in Figure 1.

For peer review only
